# Phasor-FSTM: a new paradigm for multicolor super-resolution imaging of living cells based on fluorescence modulation and lifetime multiplexing

**DOI:** 10.1038/s41377-024-01711-y

**Published:** 2025-01-03

**Authors:** Luwei Wang, Yue Chen, Jiaqing Guo, Xiaoyu Weng, Wei Yan, Jun Song, Tong Ye, Junle Qu

**Affiliations:** 1 Center for Biomedical Optics and Photonics & College of Physics and Optoelectronic Engineering, Key Laboratory of Optoelectronic Devices and Systems of Ministry of Education and Guangdong Province, Shenzhen University, Shenzhen, 518060 China; 2 The Photonics Center of Shenzhen University, Shenzhen University, Shenzhen, 518060 China; 3 Department of Bioengineering, CU-MUSC Bioengineering Program, Clemson University, Charleston, South Carolina 29634 USA

**Keywords:** Super-resolution microscopy, Biophotonics

## Abstract

Multicolor microscopy and super-resolution optical microscopy are two widely used techniques that greatly enhance the ability to distinguish and resolve structures in cellular imaging. These methods have individually transformed cellular imaging by allowing detailed visualization of cellular and subcellular structures, as well as organelle interactions. However, integrating multicolor and super-resolution microscopy into a single method remains challenging due to issues like spectral overlap, crosstalk, photobleaching, phototoxicity, and technical complexity. These challenges arise from the conflicting requirements of using different fluorophores for multicolor labeling and fluorophores with specific properties for super-resolution imaging. We propose a novel multicolor super-resolution imaging method called phasor-based fluorescence spatiotemporal modulation (Phasor-FSTM). This method uses time-resolved detection to acquire spatiotemporal data from encoded photons, employs phasor analysis to simultaneously separate multiple components, and applies fluorescence modulation to create super-resolution images. Phasor-FSTM enables the identification of multiple structural components with greater spatial accuracy on an enhanced laser scanning confocal microscope using a single-wavelength laser. To demonstrate the capabilities of Phasor-FSTM, we performed two-color to four-color super-resolution imaging at a resolution of ~λ/5 and observed the interactions of organelles in live cells during continuous imaging for a duration of over 20 min. Our method stands out for its simplicity and adaptability, seamlessly fitting into existing laser scanning microscopes without requiring multiple laser lines for excitation, which also provides a new avenue for other super-resolution imaging technologies based on different principles to build multi-color imaging systems with the requirement of a lower budget.

## Introduction

The living of cells relies on dynamic molecular activities occurring within various subcellular structures or organelles that are constantly interacting with each other. Recording the spatiotemporal sequence of these interactions is key to understanding the life process and disease development^1–3^. Therefore, the ability to simultaneously image multiple molecules or subcellular structures is crucial for such studies. The prevailing strategy of imaging multiple molecular components still relies on fluorescent labeling with multiple fluorophores, which often requires multiple laser lines for excitation and multiple spectral detection channels for recording^4,5^. As such, multicolor microscopy, also known as multiplex imaging or multichannel imaging, is in great demand in technological development. It facilitates the simultaneous visualization of multiple targets through using distinct fluorophores or fluorescent labels, significantly assisting in exploring intricate biological processes by permitting researchers to observe numerous components or interactions within a single sample^6–8^. However, emission spectral overlap between multiple fluorophores can be a challenging problem, though solvable by employing high-resolution spectral filtering or spectrometers or unmixing algorithms for separating different fluorophores; these solutions add complexity and cost to either instrumentation or imaging process. On the other hand, super-resolution microscopy (SRM) is often necessary to unveil subcellular structures and to localize specific molecules involved in physiological functions^9–12^. To perform multi-color super-resolution imaging presents a formidable challenge.

Drawing upon the principles of fluorescence on-off between energy states and/or molecule localization, a variety of super-resolution optical microscopy (SRM) techniques have been proposed in recent decades, such as stimulated emission depletion (STED) microscopy^13–15^, structured illumination microscopy (SIM)^16–18^, single-molecule localization microscopy (SMLM)^19–21^, minimal emission fluxes (MINFLUX)^22–25^, and their derivatives. To perform multicolor super-resolution microscopy with those approaches is difficult. The problems associated with multiple excitation laser lines and detection channels become more drastic, especially when the number of channels increases. For example, to realize super-resolution imaging, STED microscopy generally needs two lasers (one for excitation and the other for depletion) for each detection channel. Sharing the same depletion laser, designing and aligning two color STED is perhaps manageable; however, STED microscopy with more than two detection channels becomes challenging.

As a super-resolution microscopic imaging technique, subtraction microscopy has evolved into several variants, including fluorescence emission difference (FED) microscopy^26,27^ and intensity-weighted subtraction microscopy (IWSM)^28,29^. FED was the first proposed subtraction microscopy technique, utilizing a combination of Gaussian and doughnut beams at the apparatus level to perform subtraction with minimal intensity loss. IWSM is a well-cited method that improves resolution and reduces image distortions, compatible with various optical microscopy techniques. Using a similar idea, fluorescence spatiotemporal modulation (FSTM) technology has been recently proposed as an innovative approach to achieve super-resolution imaging using a pulsed laser with an output power of microwatt^30^. Compared to traditional subtraction microscopes, FSTM eliminates pixel mismatch caused by sample drifts in subtraction microscopy techniques, resulting in high-quality super-resolution imaging. FSTM share similar advantages over STED microscopy with a lower budget, lower illumination intensity, and fewer constraints on the choice of fluorophores.

In this paper, we present a novel multi-color super-resolution imaging method, phasor-FSTM, based on the FSTM technique. This method exploits fluorescence lifetime to encode each detection channel and utilizes phasor analysis to separate multiple components simultaneously. Specifically, the implementation of multicolor imaging is based on labeling multiple structural components with dyes that have similar excitation and emission spectra but distinct lifetimes. Due to similar excitation and emission spectra, those dyes can be excited with a single laser line and detected with a single emission channel. Implementing on a laser scanning confocal microscope, phasor-FSTM uses a time-correlated single photon counting (TCSPC) system to tag each photon’s arrival time and then phasor analysis to accurately assign each photon to a specific structural component according to the lifetime. The super-resolution is implemented by the same TCSPC system with the FSTM technique. As a result, the entire multi-color super-resolution microscope is significantly simplified, utilizing a single-wavelength pulsed laser for excitation, a photomultiplier tube (PMT) detector paired with its corresponding filter as the detection channel, and, naturally, a TCSPC unit serving as a key component for lifetime measurement and super-resolution imaging.

While our method can operate with just one excitation laser, it can also leverage multiple lasers for enhanced structural recognition through spectral separation strategies, providing greater sensitivity. Another feature of phasor-FSTM is its ability to distinguish relative lifetimes among fluorophores without knowing their specific lifetime values, which usually requires fitting procedures. Compared to existing multicolor SRM methods, our scheme significantly reduces photodamage to live cells, imposes fewer constraints on the choice of fluorophores, and importantly, overcomes current restrictions of requiring different techniques for super-resolution and multicolor imaging separately. This comprehensive scheme greatly enhances the versatility and practicality of multicolor super-resolution microscope systems, enabling the deciphering of intricate organelle interactions in substance transport and organelle homeostasis.

## Results

### Implementation of Phasor-FSTM

**Fig. 1:**
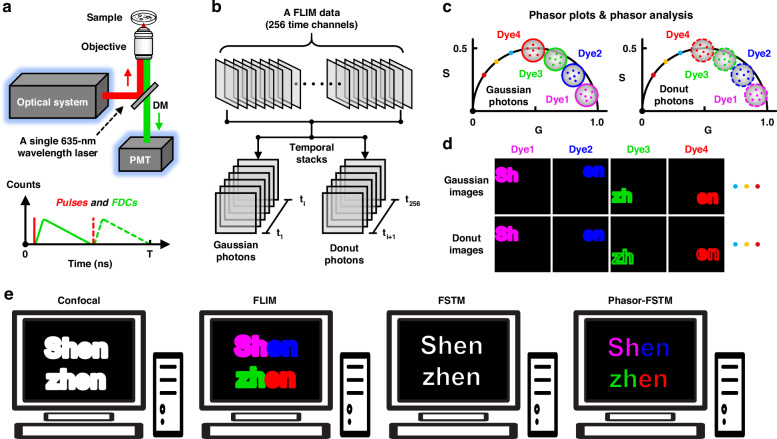
Operating principle of Phasor-FSTM method. **a** Schematic diagram of the optical system, and FLIM imaging in Phasor-FSTM mode. Pulses: Gaussian laser pulse (red solid line) and donut laser pulse (red dashed line); FDCs: Fluorescence decay curves; T: laser pulse period. **b** Separation of the FLIM data in time channels to obtain two datasets, each containing a complete fluorescence decay excited by the Gaussian and donut beams. **c** Separate phasor analysis on the two datasets to unmix the lifetime of each of the multiple components. **d** Spatial modulation to remove the diffraction-limited signals from Gaussian photons to achieve super-resolution. **e** Comparison of confocal, FLIM, FSTM, and Phasor-FSTM imaging. Confocal: diffraction-limited and structure indistinguishable; FLIM: diffraction-limited and structure distinguishable; FSTM: super-resolution and structure indistinguishable; Phasor-FSTM: super-resolution and structure distinguishable

The workflow of our phasor-FSTM method is described in Fig. 1a; see Fig. S1 and the Supplementary Information for more details. In the setup, we rely on a conventional laser scanning confocal microscope equipped with a single 635-nm pulsed laser that is divided into three parallel and independent laser beams, which are used as the reference signal for time-resolved detection and the light sources with Gaussian- and donut-shaped wavefronts for excitation, respectively. In phasor-FSTM, a sample is scanned with Gaussian and donut laser beams that are temporally delayed (Fig. S2a) but spatially overlapped (Fig. S2b). All collected photons from the PMT are recorded by a TCSPC module that builds up a photon distribution over the arrival time of the photons in the laser pulse period and the scan coordinates (Fig. S2c). The workflow for our phasor-FSTM regarding the collection and analysis of the FLIM data is shown in Fig. 1a–d. Data analysis is carried out in three steps: (1) separating the FLIM data in time channels to obtain two datasets, containing a complete fluorescence decay excited by either the Gaussian or donut laser beams; (2) performing phasor analysis on the two datasets to unmix each component according to its lifetime; and (3) removing the diffraction-limited signals from Gaussian photons to achieve super-resolution.

Before phasor-FSTM imaging, it is first necessary to select suitable fluorescent dyes (with similar spectra but distinct lifetimes), where specific and simultaneous labeling of multiple targets is extremely important. After the biological sample is labeled with the selected fluorescent dyes, FLIM imaging is performed under the illumination of the Gaussian- and donut-shaped beams alternately. To analyze FLIM data, the initial step involves determining the boundary for temporal separation. Relative to the reference pulse, the donut laser pulse follows the Gaussian laser pulse at a time delay that allows the Gaussian laser-excited molecules to return fully to the ground state, which also divides the whole decay curve into two parts in the laser pulse period. Therefore, the time channel where the peak of the donut pulse is located is treated as the boundary. It remains constant after the optical system is set up (i.e., the time interval between the two laser pulses is fixed). By using an algorithm, the FLIM file provided by TCSPC is split into two datasets (temporal image stacks), one consisting of Gaussian and the other of donut photons (Fig. 1b). The meaning of time-resolved detection based on FLIM technology is to acquire synchronized Gaussian and donut images. Besides, we can obtain a colorized FLIM image by the fitting procedure on the first image stack, which helps to reveal more biological information.

The second step is to unmix the lifetime of multiple components by photon extraction after converting the two time-resolved datasets to the phasor space. Phasor transformation is based on the mathematics originally described by Weber^31^ to resolve heterogeneous systems, and the graphical representation as a phasor plot was introduced by Jameson et al.^32^. With the TCSPC-based FLIM technique, the histogram of the photon arrival time at each pixel of the image is transformed to Fourier space, and the decay from each pixel can hence be translated to a point in the phasor plot; see Fig. S3 for more details. To ensure the accuracy and reliability of the measurement results, we tested the instrument response function (IRF) of our system from both fluorescence data and scattered signals of gold nanoparticles (Figs. S4 and S5). Note that a priori knowledge of the lifetime decay is necessary when using the time-domain FLIM technique^33^. In the phasor transformation, the photon coordinates in the phasor space can be precisely located by providing a specific lifetime value. The lifetime value and fitted fluorescence decay curve are obtained by a fitting procedure using fluorescent beads labeled with dyes commonly accepted as lifetime standards, as shown in Fig. S6. By using the same imaging configuration, subsequent data analysis does not need to provide precise lifetime information, such as fluorescence lifetime, fluorescence decay curve, and so on. We also performed additional analyses to demonstrate the robustness of our method to variations in lifetime values. By imaging HeLa cells labeled with three dyes and analyzed in confocal-FLIM mode, we show that the general integrity of the phasor plot is unaffected by the use of distinct lifetime values, confirming the reliability of our method despite potential deviations in precise lifetime calibration (see Fig. S7 for more details).

After determining the parameters, the algorithm transforms the decay data from all pixels in the two separated image stacks to form two independent phasor plots (Fig. 1c). Based on the difference in the lifetime, the phasor plot shows a region of the photon distribution with a wide span and with multiple centers depending on the number of fluorophores. For a four-lifetime species (dyes 1 to 4), the phasor points can be divided into four parts according to their coordinates; see Fig. S8 for more details. Specifically, four images of intensities *I*_1_, *I*_2_, *I*_3_, and *I*_4_, composed of photons derived from the respective components, can be formed by extracting the phasor points located within the area of four phasor plots (usually four circles) with the phasor algorithm. The rules of the phasor plot dictate that the center and radius of these circles depend on the relative lifetime and concentration of the fluorophores. By performing photon extraction for the two temporal image stacks in phasor space, we can obtain eight images of intensities *I*_g1_, *I*_g2_, *I*_g3_, and *I*_g4_ (Gaussian images) and *I*_d1_, *I*_d2_, *I*_d3_, and *I*_d4_ (Donut images), in which *g* and *d* represent the fluorescence signal excited by the Gaussian and donut laser beams, respectively.

The last step is to obtain super-resolution images by spatial modulation between the *I*_g_ and *I*_d_ images composed of photons from the same species. Mathematical processing is carried out by subtracting the image *I*_d_ from image *I*_g_ pixel by pixel with a weight coefficient (*δ*), giving a super-resolution image: *I*_s_(*x*, *y*) = *I*_g_(*x*, *y*) - *δI*_d_(*x*, *y*). The detailed mathematical derivation to determine the weight coefficient is described in the Supplementary Information. As a result, photons at the focal periphery can be removed from image *I*_g_, retaining only photons in the center of the Gaussian focal point in image *I*_s_. Due to the pixel-by-pixel subtraction in nearly real-time, the *I*_s_ image features superior SBR. In four-color imaging, we obtain four images of intensities *I*_s1_, *I*_s2_, *I*_s3_, and *I*_s4_ through spatial modulation (Fig. 1d). As a result, a four-color super-resolution image can be presented by applying different pseudo-colors to these four images and then superimposing them. Following the above steps, multicolor super-resolution imaging can be achieved, provided that the fluorescent dyes with similar spectral characteristics but distinct lifetimes are chosen and multi-target structures are specifically labeled. Figure 1e compares confocal, FLIM, FSTM, and Phasor-FSTM methods in terms of spatial resolution and structure recognition. In conclusion, our propsoed phasor-FSTM method features super-resolution and structure distinguishable. Since the time difference between the two images (*I*_g_ and *I*_d_) in the order of nanoseconds, phasor-FSTM does not suffer from pixel mismatches in spatial modulation (more generally in subtraction methods based on successive image acquisition).

### Two-color super-resolution imaging with phasor-FSTM

We first demonstrate the use of phasor-FSTM in super-resolution imaging with two colors. In our system, lifetime information is sampled in 256-time channels and the time to collect a FLIM dataset (1024 × 1024 × 256) is 4–8 seconds, depending on the quantum yield of the fluorophores (see the Supplementary Information). To test the algorithm, we used a standard sample prepared by mixing two kinds of fluorescent beads labeled with STAR 635 P (Abberior, Germany) and Alexa Fluor 647 (Abcam, UK), respectively, both 23 nm in diameter. At a total excitation power of 13.1 μW (Gaussian laser: 5.8 μW and donut laser: 7.3 μW), FLIM imaging was carried out with the acquisition time of 8 s. We loaded a 0/π phase on the SLM to form a 3D cage-typed laser beam with minimal central intensity in three-dimensional space, which can eliminate fluorescence signals from unfocused planes, further improving imaging accuracy. Importantly, it is still a donut-shaped beam with zero central intensity in the focal plane. The power density was calculated as 4.41 kW cm^−2^ and 2.95 kW cm^−2^ for the Gaussian and donut laser beams, respectively; see Fig. S9 and the Supplementary Information for more details. Following the workflow of phasor-FSTM, two-color SRM imaging of fluorescent beads was realized, as shown in Fig. S10.

**Fig. 2:**
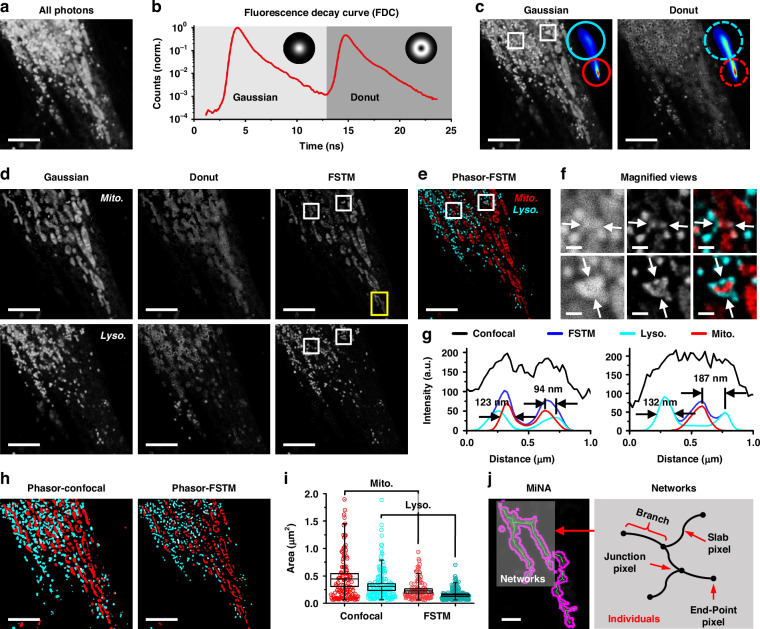
Phasor-FSTM for two-color super-resolution imaging of a living HeLa cell. **a** Intensity image of lysosomes and mitochondria composed of the photons in all time channels. **b** Fluorescence decay curve of the obtained FLIM image. **c** Gaussian and donut images obtained by temporal modulation. Inset: phasor plots and photon extraction using phasor analysis. **d** Intensity unmixing by phasor analysis to decompose the structures labeled with two dyes and excited with Gaussian and donut laser beams, and spatial modulation to achieve super-resolution (FSTM). **e** Phasor-FSTM (two-color super-resolution) image by overlapping two color-coded FSTM images in **d**. **f** Magnified views of the white squares in confocal, FSTM, and Phasor-FSTM images in **c**–**e**. **g** Intensity profiles along the arrows in **f**. **h** Object identification using adaptive thresholding on the images of Phasor-Confocal and Phasor-FSTM. **i** 2D morphological analysis of mitochondria and lysosomes in confocal and FSTM images. Data are presented as the means ± SE. **j** Example of common mitochondrial network. Networks are mitochondrial structures with at last a single node and three branches. Scale bars, 5 μm (**a, c–e, h**) and 500 nm (**f, j**)

We first demonstrated two-color phasor-FSTM with living Hela cells, where mitochondria and lysosomes were labeled with two staining kits (Invitrogen™ MitoTracker Deep Red FM, ThermoFisher Scientific; LysoBrite™ NIR, AAT Bioquest). An intensity image composed of the photons in all time channels shows a mixture of two labeled structures that are not distinguishable (Fig. 2a). The time channel where the peak of the donut pulse is located is treated as the boundary that divides the whole fluorescence decay curve (red line) into two parts (Fig. 2b). The FLIM file was split into two datasets using our phasor algorithm, and two intensity images composed of Gaussian and donut photons were obtained by summing the slides of each image stack (Fig. 2c). The phasor plots of the two image stacks were carried out after the phasor transformation and are presented in the upper-right corners of the respective intensity images. The photons were extracted to form four images, where the circles in cyan and red locate the fluorescence signal of mitochondria and lysosomes, respectively. Two phasor-FSTM images were presented at a weighted coefficient of 2 by spatial modulation (Fig. 2d). Finally, the phasor-FSTM image was realized by overlapping two color-coded FSTM images, showing two discernible structures at super-resolution (Fig. 2e). Deconvolution is employed to mitigate the blurring effects introduced by the imaging system’s point spread function (PSF), thereby further enhancing the contrast and resolution of final phasor-FSTM images; see Fig. S11 and the Supplementary Information for additional details.

Closer inspection of the enlarged confocal, FSTM, and Phasor-FSTM images from the white squares in Fig. 2c–e further proves that the phasor-FSTM method is able not only to decompose the original mixture labeled with different fluorescent dyes but also to display more details at a higher resolution (Fig. 2f). As shown in the profile plots in Fig. 2g, individual mitochondrial and lysosomal structures in Fig. 2e have the full widths at half maximums (FWHMs) of 123 nm and 132 nm, reaching one-fifth of the excitation wavelength. In addition, the resolvable distance between adjacent structures reaches 94 nm. For multiplexed analysis of the state/function of organelles, we used two ImageJ plug-ins: the Mitochondria Analyzer and the Mitochondrial Network Analysis (MiNA) toolset^34,35^. We performed object identification using adaptive thresholding on the images of Phasor-Confocal and Phasor-FSTM (Fig. 2h). In confocal and FSTM modes, the number of identified objects (mitochondria and lysosomes) is 119/110 and 155/146, with total areas of 58.74/51.49 μm^2^ and 26.77/23.33 μm^2^, respectively. The areas of each identified object in Fig. 2h and their mean areas were measured by 2D morphological analysis (Fig. 2i). The values measured in FSTM image were smaller compared to those in confocal image, suggesting higher spatial resolution, which allows for more precise segmentation of individual organelles. We further analyzed the morphology of the mitochondrial network using the MiNA toolset. Networks are mitochondrial structures with at least a single node (junction pixel) and three branches (including the end-point pixel and slab pixel). We selected the region marked by a yellow rectangle in the FSTM image of the mitochondrial structure in Fig. 2d for display, where the mitochondria in shadow in Fig. 2j is an individual with a network structure. Additional details and parameter values are provided in Fig. S12 and the Supplementary Information.

**Fig. 3:**
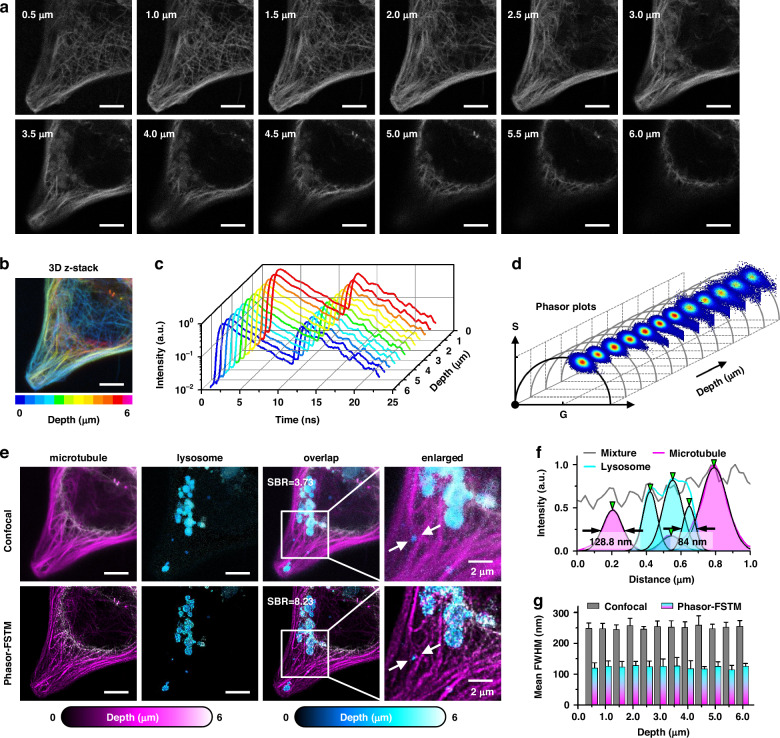
3D z-stack images of lysosomes and microtubules. **a** Unresolved confocal images at different imaging depths (z-step: 0.5 μm) in a HeLa cell. **b** 3D z-stack of confocal images in panel **a** before unmixing (different colors indicate different depths). **c** Fluorescence decay curves of the original FLIM data. **d** Phasor plots change with the imaging depth**. e** Comparison of two-color confocal and Phasor-FSTM in terms of 3D z-stack images. SBRs were calculated to demonstrate the improvement in image quality of the proposed method compared to confocal. **f** Normalized intensity profiles along the arrows in the enlarged images of **e**. **g** Mean FWHM values of the intensity profiles across the structures at five positions. Scale bars, 5 μm (**a, b, e**)

We further demonstrated two-color phasor-FSTM by imaging lysosomes and microtubules in live HeLa cells using another two kits (TraKine™ Pro Live-cell Lysosome Staining Kit, Abbkine; Cell Navigator™ Live Cell Tubulin Staining Kit, AAT Bioquest). We acquired images at different depths (Fig. 3a) and produced a 3D z-stack image with pseudo-color (Fig. 3b). Two labeled structures cannot be distinguished by the naked eye from the intensity images. Figure 3c shows the fluorescence decay curves of those FLIM data, and similar decay trends do not provide much more information about the lifetime. However, the phasor plots present obvious changes where the region occupied by the phasor points migrates to the short lifetime, which means that there are two fluorophores with different lifetimes, and their concentration ratio varies with depth (Fig. 3d). After processing the data, the organelles were resolved at different imaging depths. With increasing imaging depth, lysosomal structures emerge from nothing, which is indistinguishable from the original intensity images (Fig. 3e). The 3D z-stack image in phasor-FSTM had a higher resolution and SBR than that in confocal (8.23 for phasor-FSTM, and 3.73 for confocal). The effect was also clearly visible in the profiles of normalized intensity (128.8/107.8/168 nm for microtubules and 98.1/136.9/84 nm for lysosomes, Fig. 3f), which are marked by the white arrows in Fig. 3e. Note that the selected regions for photon extraction were fixed at different imaging depths; see Supplementary Video 1 for details. For quantitative resolution measurement, we used the FWHM of the intensity profiles across the structures at five positions at each imaging depth. Mean FWHMs of ~0.40*λ* and ~0.19*λ* were obtained in the confocal and phasor-FSTM images, respectively, and they maintain steady at different imaging depths (Fig. 3g).

### Phasor-FSTM achieves multicolor super-resolution imaging of living cells

Without adding additional optoelectronic components, phasor-FSTM can achieve multicolor super-resolution imaging in the same system, as long as multiple suitable fluorescent probes are available. To demonstrate the capability of phasor-FSTM in super-resolution imaging with more than 2 colors, we performed a series of FLIM imaging experiments in confocal mode for probe selection in terms of the lifetime and labeling specificity; see Fig. S13 and the Supplementary Information for more details. We were able to identify a set of four dyes suitable for demonstrating four-color phasor-FSTM imaging. Four commercial fluorescent probes (Invitrogen™ MitoTracker Deep Red FM and Tubulin Tracker Deep Red, ThermoFisher Scientific; LysoBrite™ NIR and Nuclear Red™ LCS1, AAT Bioquest) were selected to label specific cell organelles and structures in live cells, with their mean lifetimes of 0.3 ns, 3.3 ns, 3.8 ns, and 1.0 ns, respectively. The similar spectral properties of the four probes described above allow for simultaneous excitation and detection in the same system configuration, while their distinct lifetimes allow for multicolor imaging in phasor-FSTM. By using three of the four kits, we successfully conducted three-color phasor-FSTM super-resolution imaging (Fig. S14). After that, four organelles (lysosome, mitochondrion, microtubule, and nucleus) were labeled and imaged. Multicolor confocal imaging can be realized using phasor analysis without spatial modulation (Fig. S15).

**Fig. 4:**
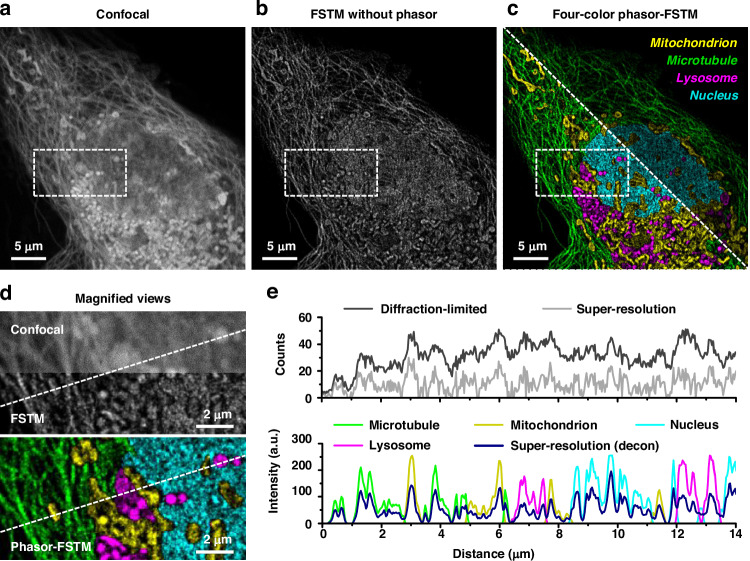
Phasor-FSTM for four-color super-resolution imaging in live cells. **a** Confocal image of a single HeLa cell consisting of the mitochondrion (MitoTracker Deep Red FM), microtubule (Tubulin Tracker Deep Red), lysosome (LysoBrite NIR), and nucleus (Nuclear Red LCS1) structures. **b** FSTM image without the phasor process. **c** Four-color phasor-FSTM super-resolution images before (top right) and after (lower left) contrast adjustment. Mitochondrion: yellow; microtubule: green; lysosome: magenta; nucleus: cyan. **d** Magnified views of the white squares in **a–c**. **e** Photon number distributions in original diffraction-limited and super-resolution images, and intensity profiles in one-color and four-color super-resolution images post-deconvolution, plotted along the dotted lines in **d**

For every pixel, the total photon number is determined by summing up the photon numbers in all-time channels, which can produce a confocal intensity image by stacking the channels that contain confocal photons (Fig. 4a). Before channel separation, the total photon count was 36,836,441, with 59.31% in the Gaussian channel and 40.69% in the donut channel. The average photon count per pixel on a 1024 × 1024 grid is 35.26 (20.84 for the Gaussian channel and 14.42 for the donut channel). Additionally, the maximum photon counts observed in pixels prior to channel separation reached 119, with post-separation peaks of 81 and 74 for the Gaussian and donut images, respectively. A super-resolution image can be obtained by using only the FSTM method (Fig. 4b). The phasor-FSTM method easily distinguishes each cellular structure with super-resolution and high SBR (Fig. 4c). With the help of high labeling specificity, we demonstrated the feasibility of our phasor-FSTM method in the multicolor imaging of four different cellular targets. The magnified views show a sharp contrast between diffraction-limited and super-resolved resolution, as well as before and after structure decomposition using phasor-FSTM (Fig. 4d). As shown in photon number distributions and intensity profiles in Fig. 4e, four subcellular structures are decomposed at super-resolution. Phasor-FSTM not only outperforms traditional multicolor imaging technologies in terms of resolution, but it also offers a versatile and feasible alternative to traditional multicolor super-resolution imaging methods based on spectral separation. Unlike these technologies, which depend on multiple laser sources and detectors, phasor-FSTM utilizes a single laser and detector, thereby achieving remarkable flexibility without compromising exceptional resolution and imaging performance.

While phasor-FSTM is less strict about dye properties than other SRM techniques such as STED, PLAM, and STORM, it still requires careful dye selection. Finding two dyes with similar spectra but distinct fluorescence lifetimes is a relatively simple task for two-color imaging. Their brightness can also be adjusted by varying the labeling concentration. However, imaging more than two structures require a set of fluorescent dyes that present similar absorption/emission spectra, distinct fluorescence lifetimes; though still manageable, the task is challenging. This endeavor necessitates extensive testing and substantial funding. Furthermore, for long-term imaging, selecting dyes that undergo photobleaching at the same rate is critical to maintain reasonable contrasts between channels. We measured the variation of the fluorescence intensity over the scanning time in phasor-FSTM mode and compared it with a confocal scheme (Fig. S16). When the laser power density is comparable, we found that the brightness decay rate of the sample in phasor-FSTM is similar to that of confocal. However, under the same lighting conditions, these dyes exhibit different excitation efficiency and fluorescence decay rate due to their unique chemical properties (see Figs. S17 and S18 for more details). Phasor-FSTM is essentially equivalent to a conventional confocal system, but with higher spatial resolution and a strong structural identification ability when combined with FLIM and fluorescence modulation techniques. Compared to those SRM techniques based on scanning imaging systems, such as STED, MINFLUX, and their derivatives, phasor-FSTM has lower requirements and fewer constraints on fluorescent dyes and sample preparation. Consequently, fluorophores with lower photobleaching resistance can be used in the scan-based optical system for long-term imaging, e.g., fluorescent proteins with higher cell affinity.

### Time-lapse phasor-FSTM imaging monitors mitochondrion–lysosome interactions

**Fig. 5:**
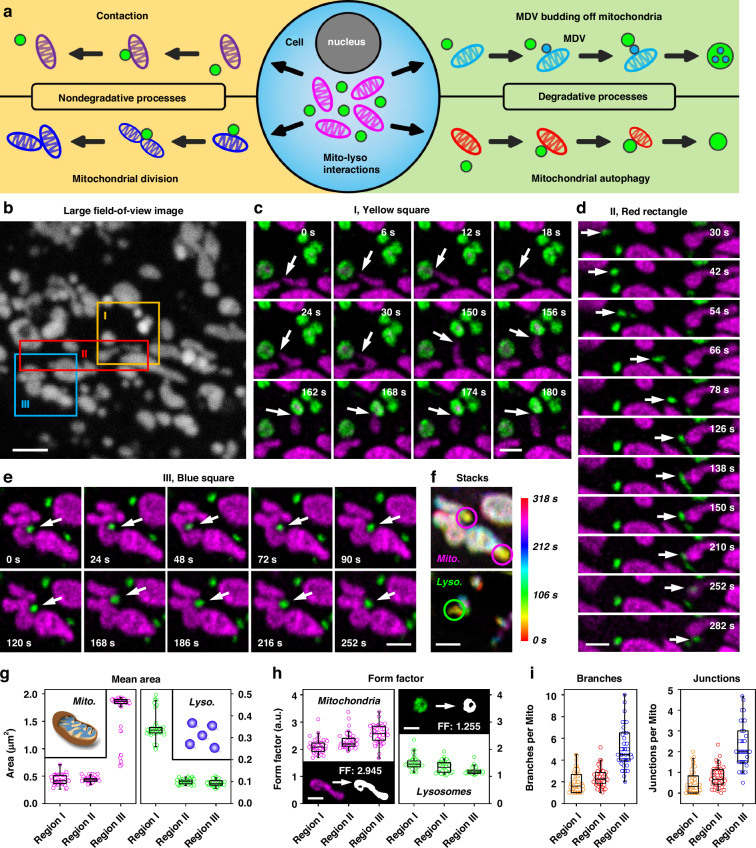
Phasor-FSTM monitors the interactions of mitochondria and lysosomes by long-term imaging of a live HeLa cell. **a** Schematic of the process of the direct interactions between mitochondria and lysosomes. **b** A large field-of-view intensity image of mitochondria and lysosomes (without unmixing) at one time point during their mutual interactions. **c–e** Time-lapse phasor-FSTM images reveal different dynamic physical interactions between mitochondria and lysosomes in three marked regions in **b**. **f** Time-color-coded phasor-FSTM stack images in **e** (different colors indicate different imaging times). Magenta and green circles denote the moment when the lysosome pushes the mitochondria into maximum deformation. **g** Mean areas of mitochondrial and lysosomal structures in the three regions in 54 phasor-FSTM images. **h** Form factors of mitochondrial and lysosomal structures in the three regions in 54 phasor-FSTM images. Insets: Phasor-FSTM images of individual mitochondrion (bottom left) and lysosome (top right) and their adaptive threshold images with form factors of 2.945 and 1.255, respectively. **i** Branches and junctions of mitochondrial structure in the three regions in 54 phasor-FSTM images. Data are presented as the means ± SE. Scale bars, 2 μm (**b**) and 1 μm (**c**–**f, h**)

Mitochondria and lysosomes are two vital organelles within biological cells, and their interactions play a crucial role in cellular activities. Previous studies have demonstrated that these organelles can engage in direct interactions through both degradative and non-degradative processes^36–38^. Lysosomes degrade mitochondria through either mitophagy or the fusion of mitochondrial-derived vesicles (MDVs) with lysosomes. Both mechanisms are crucial for eliminating impaired mitochondria, thereby promoting cellular function and survival. Meanwhile, mitochondria and lysosomes can also interact directly with one another under normal conditions in healthy mammalian cells via nondegradative pathways, which involve mitochondrion–lysosome contact sites (Fig. 5a). A series of long-term phasor-FSTM imaging experiments were performed, and abundant dynamic interactions between mitochondria and lysosomes were observed (Fig. 5, Figs. S13 and S14, and Supplementary Videos 2–8). We found that in areas where there are dense mitochondria and lysosomes, numerous interactions may be occurring simultaneously.

Fifty-four FLIM images were captured by continuous imaging (4 s/frame, with 2 s dark recovery) over a period of 318 s. Figure 5b shows a large field-of-view (FOV) intensity image of mitochondria and lysosomes (without unmixing) at one time point during their mutual interactions in a HeLa cell. Using phasor-FSTM, two subcellular structures were demodulated (green for lysosomes, and magenta for mitochondria) and presented at super-resolution (Supplementary Video 2). We focused on the regions defined by two squares and a rectangle in Fig. 5b. In the first region (yellow square), a mitochondrion made active contact with two lysosomes successively, demonstrating the process of mitophagy (each contact time < 30 s). However, it is more common for the mitochondria to remain in place while the lysosomes actively contact them (Fig. S13a and Video 3). In the second region (red rectangle), a lysosome (marked by the white arrow) breaks away from a mitochondrion on the left side of the image, then moves to the right and binds to another one (Fig. 5d). We found that the lysosome moves rapidly when it is not in contact with the mitochondria (the maximum speed is 0.257 μm/s), but slows down when it gets closer to another one; see Fig. S19 and the Supplementary Information for more details. Using phasor-FSTM at high spatial resolution, mitochondria were found to contact multiple lysosomes, and lysosomes could contact multiple mitochondria. A previous study demonstrated that lysosomes moving in proximity to mitochondria often resulted in significant changes to the mitochondrial morphology^39^. This phenomenon was also observed under phasor-FSTM (Fig. 5e and Supplementary Video 4). For example, a lysosome (marked by white arrows in Fig. 5e) pushed mitochondria into invagination. The deeper the lysosome is embedded into the mitochondria, the more obvious the mitochondrial deformation is (magenta and green circles in Fig. 5f). The increase in the contact area might accelerate physiological functions mediated by mitochondrion–lysosome contacts.

For 2D analysis, we characterize the size and the shape of the mitochondrion and lysosome, whereas the shape of structures is defined by the form factor (FF) derived as the inverse of the “circularity” output value (circularity = 0.00–1.00). The mean areas of the mitochondrial and lysosomal structures in the three regions in 54 phasor-FSTM images were calculated (Fig. 5g). Remarkably, the mitochondria of the third region and the lysosomes of the first region have a larger mean area, which is also seen in Fig. 5e, c. Due to the complex structure of the organelles, a larger mean area tends to imply a larger form factor, i.e., a more irregular shape (Fig. 5h). A mitochondrion and a lysosome in the first frame of phasor-FSTM were selected to compare the form factor. By using adaptive thresholding to identify the objects, the form factors of 2.945 and 1.255 were obtained, showing the elongated structure of the mitochondrion and the circular structure of the lysosome. We performed 2D morphological analysis of mitochondria and lysosomes on full-field phasor-FSTM images to determine the variation of mitochondria and lysosome count, total area, mean area, mean perimeter, and mean aspect ratio (AR) curves over time; see Fig. S20 and Supplementary Information for more details. Finally, we evaluated the overall connectivity and morphological complexity of the mitochondrial network based on the skeletonized network and quantified this by the number of branches and the number of branch junctions in the skeleton. In the skeletonized network, the number of branches and branch junctions progressively increased, illustrating that the complexity of the mitochondrial network is associated with the increase in the mean area (Fig. 5i).

Dynamic mitochondrial tubulation has been reported to be important for maintaining mitochondrial DNA integrity and interchanging mitochondrial material, which contributes to the dynamic remodeling of the mitochondrial network^40^. Mitochondria can protrude a thin tubule from their main body during life activities, either autonomously or by pulling lysosomes. Many of these tubules retracted back to the original mitochondrion within a minute (Fig. S21b and Supplementary Video 5). In the other case, the tubule from the first builds a bridge under the guidance of lysosomes, leading to the fusion of two adjacent mitochondria (Fig. S21c, d and Supplementary Videos 6 and 7). Under long-term phasor-FSTM imaging over 24.5 min (8 s/frame, with 22 s dark recovery), we observed multiple dynamic processes of mitochondria and lysosomes in the same FOV (Fig. S22). More details are provided in Supplementary Video 8 and the Supplementary Information.

Undoubtedly, the realization of mitochondrial and lysosomal functions also requires the help of other subcellular structures. We initially studied the interaction among three organelles with long-term three-color phasor-FSTM imaging, i.e., mitochondria (MitoTracker™ Deep Red FM), lysosomes (LysoBrite™ NIR), and microtubules (Tubulin Tracker™ Deep Red). The processes of mitochondrial fusion and tubulation, as well as MDV budding off mitochondria and hitchhiking along microtubules, were observed during the imaging course of 7 min; see Fig. S23, Supplementary Video 9 and the Supplementary Information for more details. In a word, we provide visual evidence of dynamic physical interactions over different time courses using the phasor-FSTM method. The results show that organelles interact with each other over different lengths of time to perform their functions. To the best of our knowledge, this is the first time that multicolor super-resolution imaging has been achieved using only a single-wavelength laser for excitation and a detector for detection. Taken together, our results demonstrate the tremendous potential of phasor-FSTM for biological research, especially in the study of organelle interactions and other cellular activities. We have made comparisons with conventional multicolor super-resolution fluorescence microscopy technologies (Fig. S24). Furthermore, we investigated the impact of various photon number ratios on the phasor plots in two-color imaging (Fig. S25). Phasor-FSTM, which uses only a single wavelength laser, a PMT detector, and a TCSPC module, allows for up to four-color super-resolution imaging, presenting a model capable of achieving simplicity, flexibility, and affordability.

## Discussion

We presented a novel super-resolution microscopy method that uses an easy-to-implement FSTM system for acquiring spatiotemporal information of photons, combined with a powerful phasor-based processing algorithm for unmixing components in FLIM datasets. By integrating fluorescence modulation with lifetime multiplexing, our method simultaneously identifies multiple structural components, offering unbiased and high signal-to-background ratio (SBR) multicolor super-resolution imaging of live cells. Based only on a regular point detector and a single pulsed laser source with a output power of microwatt, the phasor-FSTM method provides rapid FLIM data analysis, opening a new avenue for low-budget multicolor SRM systems. In addition, phasor-FSTM has the same requirements for sample preparation as confocal microscopy yet lower constraints on fluorophores than almost any other SRM technology. Therefore, phasor-FSTM is expected to transform all confocal microscopes into super-resolution microscopes and offer a reliable alternative to spectral separation strategy for multicolor imaging.

We demonstrated the applicability of phasor-FSTM with a series of live cell imaging experiments. The information obtained from these experiments allowed for simultaneous four-color SRM imaging with a single light source, leading to the direct visualization and quantification of subcellular structures with diverse biophysical signatures. Our method not only functions effectively with a single excitation laser, but also further increase the number of recognizable structures by using multiple lasers combined with spectral separation technology. In short, phasor-FSTM further pushes the limits of fluorescence microscopy for analyzing biological and biomedical specimens. In an era of information explosion, we strive to satisfy the ever-growing need for simple but powerful and stable multicolor SRM technology. Phasor-FSTM isn’t directly compatible with SIM or SMLM due to their differing imaging modes, but it could become feasible with modifications. Integrating point-scanning SIM with time-domain FLIM or SMLM with frequency-domain FLIM, followed by phasor analysis, could enable multi-color super-resolution imaging with a single-wavelength laser, significantly broadening applications.

As the number of microstructures to be observed increases, phasor-FSTM encounters limitations. Since photons from the labeled dyes are collected simultaneously, the fluorescence intensity among them should be comparable. For long-term imaging in particular, it is better to maintain a consistent photobleaching rate for the dyes, as this determines the length of effective imaging time. Therefore, the selection and use of dyes are crucial to the method, which, of course, will not present much of a problem for chemists. Additionally, it is practical to use the same dye for specifically labeling multiple structures simultaneously through the design of the material structure, and the dye has distinct fluorescent lifetimes after labeling, such as quantum dots and carbon dots^41,42^. Finally, it is worth noting that a multiplier effect can occur when implementing an extra laser source with a different wavelength and combining the spectral separation strategy. We anticipate that by integrating the phasor-FSTM method with other techniques a comprehensive structural map of the entire cell can be obtained, thereby contributing to further studies of the form, function, and mechanism of organelle interactions.

While studying organelle interactions with phasor-FSTM, we have faced a challenge concerning the analysis of multiple components within a single-pixel. Specifically, accurately separating and identifying more than two components within one pixel has been a limitation of our current technique. We recognize that this aspect represents a significant hurdle in achieving optimal performance in complex biological environments where multiple fluorescent signals may overlap. To address this limitation, we are actively pursuing a new demodulation technique that aims to precisely demodulate photons originating from different subcellular structures within a single pixel. By acknowledging this challenge and actively seeking solutions, we believe that our phasor-FSTM method as a new paradigm for multicolor super-resolution imaging in living cells will continue to evolve and ultimately enable unprecedented insights into the intricate dynamics of subcellular organization and function.

## Materials and methods

### Microscopy

All data were acquired with a home-built microscope system described in Fig. S1 and the Supplementary Information. The improved FSTM method is implemented on a conventional laser scanning confocal microscope (LSCM) that worked in tandem with a picosecond laser (LDH-D-C-635, PicoQuant, Germany), a photomultiplier tube (PMT) (H7422-40, Hamamatsu Photonics, Japan), and a TCSPC module (SPC-150, Becker & Hickl GmbH, Germany). A Leica 100×/1.40 numerical aperture (NA) oil objective was used for all experiments.

### Cell culture

HeLa cells were cultured in Dulbecco’s modified Eagle’s medium (#11965118, DMEM, ThermoFisher Scientific), supplemented with 10% fetal bovine serum (#26140079, FBS, ThermoFisher Scientific) and penicillin-streptomycin solution (100 units of penicillin and 100 μg/ml streptomycin in 0.85% saline solution, GenClone), and incubated with 5% CO_2_ at 37 °C.

### Sample preparation

A total of 2 × 10^5^ cells were seeded on a glass-bottom micro-well dish and incubated with 1 ml of DMEM supplemented with 10% FBS for 24 h. After an overnight incubation, the cells were washed three times with PBS. For three-color imaging, the cells were incubated in a 5% CO_2_ atmosphere at 37 °C for 15 min and washed by PBS for 3 times after adding 100 nM LysoBrite™ NIR (AAT Bioquest) diluted in DMEM (supplemented without 10% FBS). Followed by 200 nM MitoTracker™ Deep Red FM (Thermofisher) was used for 30 min and then the supernatant was discarded. Afterwards, the cells were incubated with 4 μM Tubulin Tracker™ Deep Red (Thermofisher) at 37 °C for another 1 h. After treatment, the cells were rinsed 3 times in a wash buffer. Finally, the cells were cultured in medium and observed under microscopy. In four-color imaging, the cells were incubated with 100 nM Nuclear Red™ LCS1 (AAT Bioquest) at 37 °C for 30 min prior to the addition of the Tubulin Tracker. To minimize sample preparation time, the cells can be incubated simultaneously with the probes used. Note that incubation in this way sometimes results in a change in the fluorescence lifetime of the dye. Fortunately, phasor-FSTM focuses not on specific fluorescence lifetimes, but on relative fluorescence lifetimes between the dyes used. Therefore, as long as the dyes have the lifetime difference after labeling the structures, the proposed method enables multi-color super-resolution imaging under the simplest sample preparation conditions/requirements.

For more information about the cellular staining reagents/kits, please visit

LysoBrite™ NIR (ex/em: 636/651 nm):

https://www.aatbio.com/products/lysobrite-nir?unit=22641

MitoTracker™ Deep Red FM (ex/em: 644/665 nm): https://www.thermofisher.cn/order/catalog/product/M22426#/M22426

Tubulin Tracker™ Deep Red (ex/em: 652/669 nm): https://www.thermofisher.cn/order/catalog/product/T34077?SID=srch-srp-T34077

Nuclear Red™ LCS1 (ex/em: 622/645 nm):

https://www.aatbio.com/products/nuclear-red-lcs1-5-mm-dmso-solution?unit=17542

## Supplementary information

Supplementary information for Phasor-FSTM: a new paradigm for multicolor super-resolution imaging of living cells based on fluorescence modulation and lifetime multiplexing

Supplementary Video 1

Supplementary Video 2

Supplementary Video 3

Supplementary Video 4

Supplementary Video 5

Supplementary Video 6

Supplementary Video 7

Supplementary Video 8

Supplementary Video 9

## Data Availability

The data that support the findings of this study are available from the corresponding author upon reasonable request. The code and test data are provided in this paper.
