# Supplementary material for: Phasor-FSTM: a new paradigm for multicolor super-resolution imaging of living cells based on fluorescence modulation and lifetime multiplexing
Source: Light Sci Appl. 2025 Jan 3;14:32. doi: 10.1038/s41377-024-01711-y (PMC11697263; doi:10.1038/s41377-024-01711-y)
Supplement: Supplementary file 1 — Supplementary information for Phasor-FSTM: a new paradigm for multicolor super-resolution imaging of living cells based on fluorescence modulation and lifetime multiplexing [file 41377_2024_1711_MOESM1_ESM.pdf]

Supplementary information

Phasor-FSTM: a new paradigm for multicolor super-resolution imaging of living cells based on fluorescence modulation and lifetime multiplexing

Luwei Wang,[1,†] Yue Chen,[1,2,†] Jiaqing Guo,[1] Xiaoyu Weng,[1] Wei Yan,[1] Jun Song,[1,*] Tong Ye[3,*] and Junle Qu[1,*]

[1]Center for Biomedical Optics and Photonics & College of Physics and Optoelectronic Engineering, Key Laboratory of Optoelectronic Devices and Systems of Ministry of Education and Guangdong Province, Shenzhen University, Shenzhen 518060, China

[2]The Photonics Center of Shenzhen University, Shenzhen University, Shenzhen 518060, China

[3]Department of Bioengineering, CU-MUSC Bioengineering Program, Clemson University, Charleston, South Carolina 29634, USA

[†]These authors contributed equally: Luwei Wang, Yue Chen.

[*]Corresponding author: jlqu@szu.edu.cn, ye7@clemson.edu, songjun@szu.edu.cn

Supplementary Information

In this supplementary material, we describe the microscope and the data processing used in our work that is relevant to our implementation of Phasor-FSTM

Microscope Setup

The recently proposed FSTM method has achieved super-resolution imaging at a resolution higher than 100 nm. It is implemented on a laser scanning confocal microscope (LSCM) with a pulsed diode laser that has a pulse width of ~100 ps and a frequency of 80 MHz [1]. In the original scheme, the output of the laser beam was divided into two excitation beams: one was converted to a donut-shaped wavefront by a vortex phase plate, and the other was unmodulated and remained as a Gaussian beam. Pulses of the Gaussian beam were optically delayed by ~2 ns relative to those of the donut-shaped beam, resulting in the fluorophore being excited twice in within a pulse repetition cycle of 12.5 ns. To reduce the potential damage of laser to biological samples and the decay rate of fluorescence intensity when performing long-term SRM imaging, we presented an improved FSTM scheme that tuned the pulse rate to 40 MHz and expands the time delay (between two laser pulse trains) by a value between twice the fluorescence lifetime of the fluorophore and half a pulse period. In this case, the decay curve collected by TCSPC-based time-domain electronics setup can collect two complete processes of spontaneous emission for conventional probes whose lifetime is typically a few nanoseconds. Furthermore, the sequence of two laser pulse trains is irrelevant. The benefit of this is that the excitation frequency is reduced by half, which is essential for maintaining a high signal-to-background ratio (SBR) of SRM images in continuous, long-term imaging.

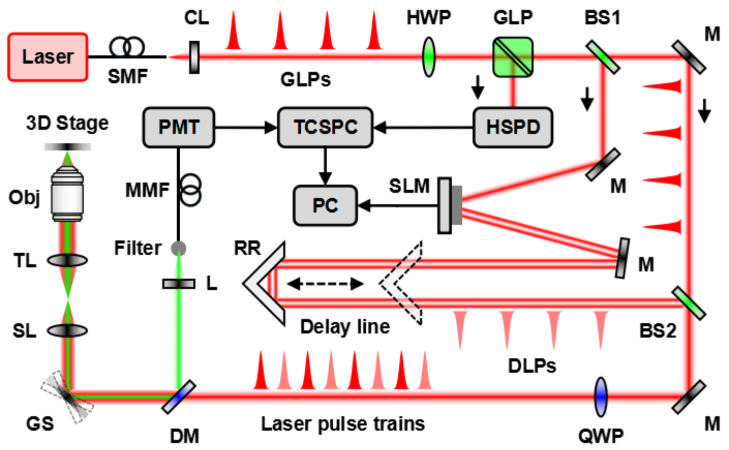

Figure S1 Schematic depiction of the home-built microscope setup. SMF, polarization-maintaining single-mode fiber; CL, collimating lens; HWP, half-wave plate; GLP, Glan-laser polarizer; BS, beam splitter; M, mirror; SLM, spatial light modulator; RR, retro reflector; QWP, quarter-wave plate; GS, galvanometer scanner; SL, scan lens; TL, tube lens; Obj: Objective; DM, dichroic mirror; L, achromatic lens; MMF, multimode fiber; HSPD, high-speed photodiode detector; PMT, photomultiplier tube; TCSPC, time-correlated single photon counting; PC, personal computer; GLPs, Gaussian laser pulses; DLPs, donut laser pulses

The FSTM method is implemented on a conventional laser scanning confocal microscope (LSCM) that worked in tandem with a picosecond laser (LDH-D-C-635, PicoQuant, Germany) and a photomultiplier tube (PMT) (H7422-40, Hamamatsu Photonics, Japan), as shown in Fig. S1. After emerging from a polarization-maintaining single-mode fiber (SMF), a 40 MHz pulsed laser was collimated and divided

into three beams when it passes through a Glan-laser polarizer (GLP) and a beam splitter (BS1). One beam reflected by the GLP is detected by a high-speed photodiode detector (HSPD) (PHD-400, Becker & Hickl GmbH, Germany) as a reference signal for time-resolved detection. The remaining two beams were used for illuminating the sample, one of which was transmitted to a spatial light modulator (SLM) (PLUTO-NIR-011, HOLOEYE Photonics AG, Germany). The SLM enables aberration correction of the laser beam for deeper imaging depth and better image quality in biological imaging, which loads a 0~2π vortex phase in the middle of the liquid crystal display to generate a donut-shaped focal spot and enables aberration correction of the laser beam for deeper imaging depth and better image quality in biological imaging. The laser incident on the SLM is rendered to *p*-polarized so that it can be fully modulated, then converted to circular polarization by a quarter-wave plate (QWP) before entering the objective (HCX PL APO, 100×/1.40-0.70 OIL, Leica, Germany). The output beam from the SLM is spatially overlapped with the Gaussian beam at the second beam splitter (BS2) and imaged onto a pair of galvanometers (6210H, Cambridge Technology, USA), which is in turn conjugated with the pupil of an oil immersion objective. A scan lens (SL) and a tube lens (TL) are implemented after the scanner to expand the diameters of the combined beams to take full advantage of the numerical aperture. The sample is fixed on a three-dimensional (3D) stage (MPC-385 Series, Sutter Instrument, USA) with a displacement accuracy at the nanoscale. The fluorescence signal emitted from the sample is collected by the objective, de-scanned by the scanner, and reflected by the dichroic mirror (DM) (ZT647rdc, Chroma, USA). After being focused by a lens (LBTEK, China) and filtered through a bandpass filter (ET667/30m, Chroma, USA), the fluorescence is coupled into a multi-mode fiber (MMF), which functions as a pinhole with a size of 1.0 Airy units, and subsequently collected by the PMT. The donut-shaped and Gaussian beams are derived from the same laser so that they have same pulse rate, and their pulse trains are precisely synchronized when the optical path of the system is fixed. Significantly, the donut beam is fed into a movable retro reflector (RR) (PS976M-B, Thorlabs, USA) so that it has a longer optical path than the Gaussian beam when reaches the sample. Finally, both the fluorescence and reference signal are transmitted to a time-correlated single photon counting (TCSPC) module (SPC-150, Becker & Hickl GmbH, Germany), which records, for each photon, the absolute arrival time and the relative time to the reference signal from the excitation laser beams and then builds up the spatial and temporal distributions of the photons.

Spatiotemporal Relationship of Two Excitation Lasers

In phasor-FSTM imaging, a sample is scanned by Gaussian and donut laser beams, which are temporally delayed but spatially overlapped (**Figure S2**). Since the optical path passed by the Gaussian laser is shorter than that of the donut laser, the fluorescence produced under its excitation is first detected by the PMT. Given that the time delay between two laser pulse trains is much larger than the fluorescence lifetime of the dye, the decay curve collected by a TCSPC module involves two complete processes of spontaneous emission in one pulse period (**Figure S2**, **a**). Based on the principle of super-resolution imaging of compressing the point spread function (PSF), the Gaussian and donut focal spots must be highly overlapped in space. Indeed, this is the foundation for acquiring high-quality phasor-FSTM images. The scatter images of 150 nm Au nanoparticles were used as a criterion, and the spatial overlap was adjusted by intensity imaging in real time (**Figure S2**, **b**). All collected photons from the PMT are recorded by a TCSPC module that builds up a photon distribution over the arrival time of the photons in the laser pulse period and the scan coordinates (**Figure S2**, **c**). In our system, the signal is sampled in 256 channels in one pulse period, and the time decay is greater than 10 ns.

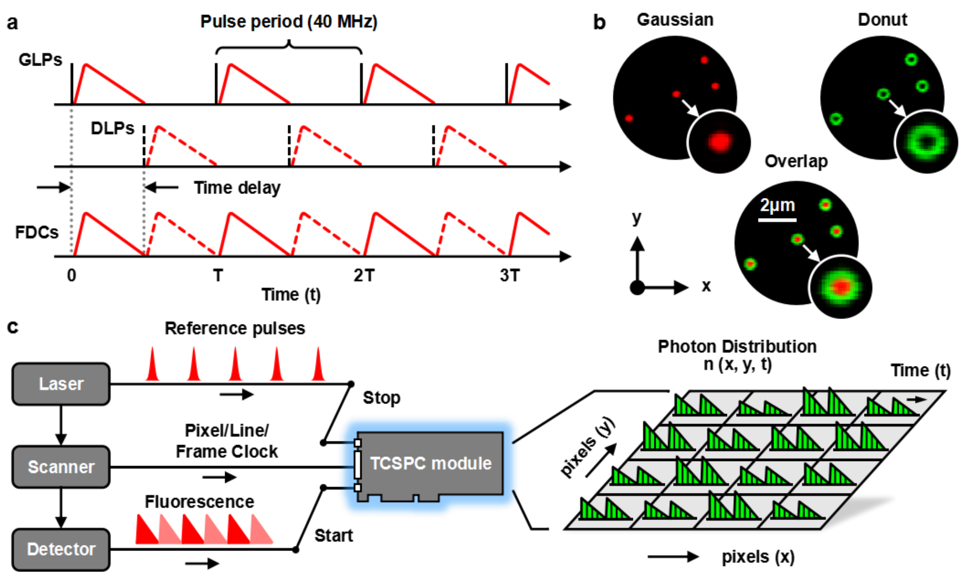

Figure S2 a Temporal relationship between Gaussian and donut laser pulses. GLPs, gaussian laser pulses; DLPs, donut laser pulses; FDCs, fluorescence decay curves. **b** Spatial relationship between Gaussian and donut laser beams. **c** Principle of TCSPC-based FLIM imaging in the phasor-FSTM method

Fluorescence lifetime multiplexing based on phasor plot analysis

Phasor Transformation

Through the extension of confocal, FSTM is already an SRM technique that is easy to implement and use. However, the large amount of data and the inevitable overlapping of spectral emissions from multiple species require rapid and precise analysis so that the method is more broadly applicable and accessible. Due to its ease of implementation, speed, and the immediate graphical representation of FLIM data, the phasor approach for the analysis of FLIM datasets was introduced over a decade ago and has found extensive applications [2–5]. Recently, the approach has been extended to the analysis of spectral signals [6–9]. Data processing exploits a phasor-based algorithm that relies on the analysis and unmixing of the lifetime phasors for each of the components to obtain the pure lifetime signature of the species. To obtain an accurate lifetime value by fitting the fluorescence decay curve, FLIM images typically need at least hundreds or thousands of photons per pixel. This results in a slow imaging rate and, consequently, low temporal resolution. When addressing this problem by shortening the acquisition time (i.e., fewer photons), undersampling occurs, resulting in a bias in the fitting procedure that skews the results. However, this bias is much less prominent if the data are analyzed with the phasor approach, which is also slightly more precise and faster [6].

Phasor FLIM provides a 2D graphical view of lifetime distributions. This graphical view enables any observer to distinguish and separate different lifetime populations within a FLIM image rapidly. Multiple molecular species are resolved within a single pixel, because every species has a specific phasor. In FLIM imaging, the decay times of fluorophores are determined at each spatial location of an image. Assuming an infinite excitation pulse, an intrinsic fluoresce decay composed of N ($N \geq 1$) fluorescent species with distinct fluoresce lifetimes can be modeled as follows:

$$I(t) = I(0)\sum_{i=1}^{N}\alpha_i e^{-t/\tau_i} \tag{1}$$

where $I(0)$ is the number of the instantly emitted photons at time zero; the coefficient α_i, called the pre-exponential factor, is the amplitude; and τ_i is the fluorescence decay time of the *i*-th component of the mixture. In TCSPC-based FLIM, each pixel location recorded a decay trace $I(t)$ within the pulse period, which was plotted as a phasor point with the coordinates (g, s) in the phasor plot after the sine and cosine transforms, as shown by Equation (2) and Fig. S3a:

$$g(\omega)=\frac{\int_0^\infty I(t)cos(\omega t)}{\int_0^\infty I(t)}, \quad s(\omega)=\frac{\int_0^\infty I(t)sin(\omega t)}{\int_0^\infty I(t)} \tag{2}$$

where the modulation frequency ω denotes the laser repetition angular frequency, which is calculated by multiplying the laser repetition rate by 2π. Furthermore, the photons illuminated by the excitation laser can be represented in polar coordinates (m, φ) through the frequency-domain (FD) method. In the FD method, the two coordinate values, m and φ, represent the amplitude change and phase delay relative to the excitation laser, respectively. Since the TCSPC and FD data can be interconverted by Fourier transform, the two methods are mathematically equivalent. Therefore, the polar coordinates (m, φ) can be mapped into the phasor plot, as shown in Equation (3) and Fig. S3b:

$$g(\omega)=m\times cos(\varphi), \quad s(\omega)=m\times sin(\varphi) \tag{3}$$

For a single-lifetime species (N = 1), the phasor coordinates are related to the fluorescence lifetime (τ) and modulation frequency (ω):

$$g=\frac{1}{1+\omega^2\tau^2}, \qquad s=\frac{\omega\tau}{1+\omega^2\tau^2}, \qquad \tau=\frac{1}{\omega}\left(\frac{s}{g}\right) \tag{4}$$

These define a semicircle (referred to as a “universal semicircle”) centered at (0.5, 0) with radius 0.5 in the phasor space with Equation (5), as shown in Fig. S3b:

$$(g-0.5)^2+s^2=0.5^2 \tag{5}$$

In the phasor transformation, each pixel in the image is plotted as a single point in the phasor plot by applying the sine and cosine transforms to the measured decay data (**Figure S3**, **c**). This is equivalent to the real and imaginary components of the Fourier transform of the decay data [2].

We coupled the improved FSTM system with a phasor-based processing algorithm meant to provide fit-free analysis and unmixing of the FSTM dataset for multicolor super-resolution imaging. Therefore, the main advantage of phasor-FSTM is that it is based on the unbiasedness of the phasor approach. In addition, our strategy for achieving multicolor imaging is to take advantage of the differences in the fluorescence lifetime. Without fitting the lifetime decay curve and even without a priori knowledge of specific lifetime values, the phasor approach can provide a graphical global view of fluorescence decay at each pixel in the phasor space [10–13]. Therefore, the difference in the fluorescence lifetime is crucial to determining the number of components that can be separated and the degree of separation between them. Depending on the pulse rate of the laser and the fluorescence lifetime of the dye used (typically in the order of nanoseconds), a difference of at least a few hundred picoseconds is required. In short, the phasor approach increases precision and decreases bias, making it ideal for analyzing FLIM datasets. Without adding additional optoelectronic components, a confocal system combining fluorescence modulation and lifetime multiplexing enables low-power multicolor super-resolution

imaging of live cells over long periods of time using only a single-wavelength laser.

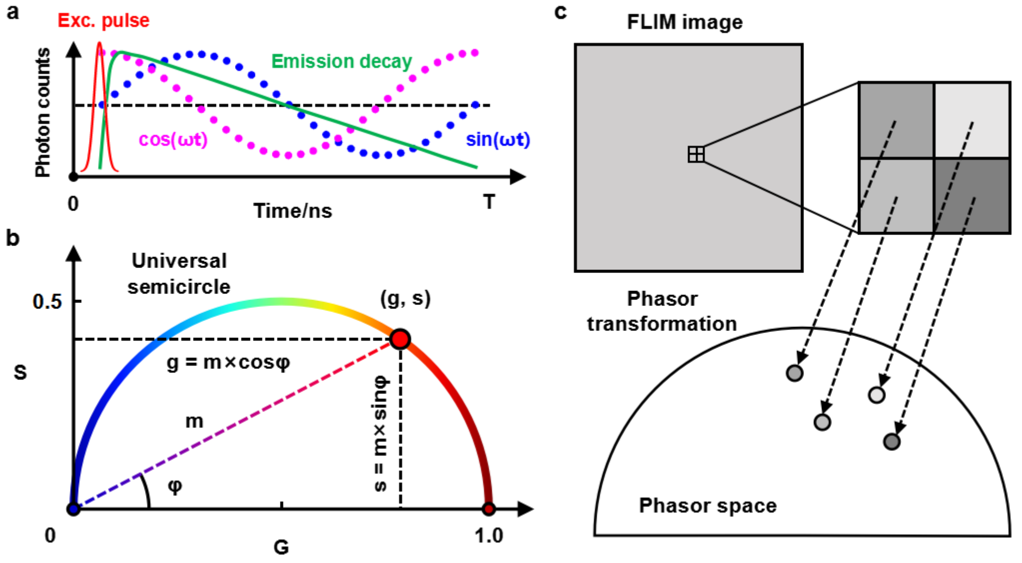

Figure S3 a TCSPC-based time-domain FLIM technique. **b** Phasor plot with the "universal semicircle" illustrated. **c** Phasor transformation from the FLIM image (spatial domain) to the phasor space (frequency domain)

Calculation of IRF

In practical applications, the recorded IRF may not always be accurate due to factors such as light scattering and reflection [14,15]. However, SPCImage software has functions to derive the IRF from recorded FLIM data [15]. The IRF can be obtained from the fluorescence signal: the calculation procedure fits the rising edge of the fluorescence signal with a suitable function, Rise(t). The differentiated function, d Rise(t) / dt, is the IRF. Figure S4 illustrates this process and shows a typical result obtained using this method.

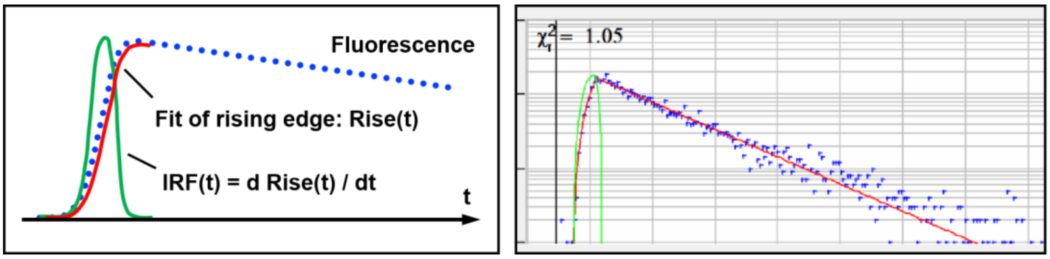

Figure S4 Calculation of IRF from fluorescence data (principle: left; typical result: right)

Furthermore, as a direct approach to measure the system IRF, we performed scattering imaging of Au nanoparticles (see **Figure S5**) using confocal imaging mode without a laser-blocking filter. This configuration primarily collects scattered laser light. By selecting an appropriate spot and adjusting the binning factor, we were able to derive the IRF from the chosen area's waveform (**Figure S5**, middle). Figure S5, on the right, shows the result of clicking the "copy IRF from decay data" button. The green curve represents the IRF, and Gaussian fitting yields a full width at half maximum (FWHM) of 186.43 ps. Considering the effect of scattered or reflected light, the actual system IRF should be smaller. This value is lower than the minimum fluorescence lifetime of the dyes used in our experiments, indicating our method's ability to accurately measure fluorescence decay. In two-photon microscopes a measured IRF is usually obtained from second harmonic generation (SHG) data.

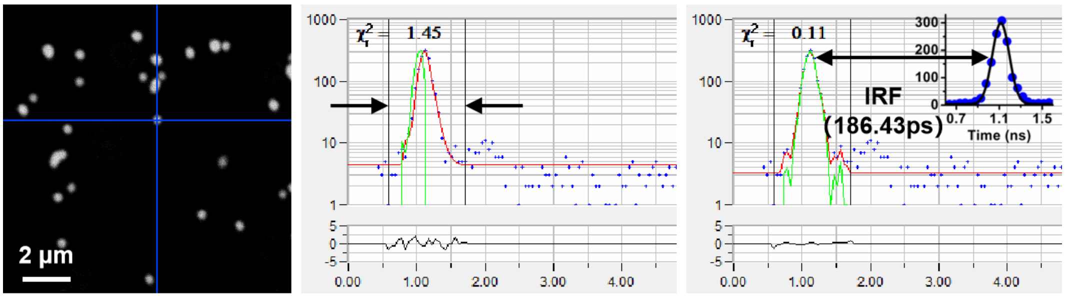

Figure S5 Calculation of IRF from scattered signals of gold nanoparticles. Left: Scattering image recorded from Au nanoparticles. Middle: Waveform in selected spot of the image. Large binning used, cursors pulled to the beginning and the end of the pulse. Right: IRF after clicking the ‘copy IRF from decay data’ button on the software interface (SPCImage 8.8)

<u>Parameter determination of the phasor algorithm</u>

When using the time-domain FLIM technique, a priori knowledge of the lifetime decay has to be taken into consideration. In the phasor transformation, the photon coordinates in the phasor space can be precisely located by providing a specific lifetime value. These parameters are determined by a fitting procedure that involves a standard sample with a well-known lifetime, usually a fluorescent beads sample labeled with a commercial dye. However, in multicolor imaging, if we ignore the information in the lifetime dimension and only focus on the physical contacts between the structures to be observed, the specific lifetime values of the dyes used do not necessarily need to be provided. At this point, it is only necessary to ensure that the lifetime of fluorescent dyes labeled to the structures is significantly different. Therefore, our phasor algorithm requires only three parameters: a data trace, a fit curve, and a lifetime value, which are already provided during the system calibration.

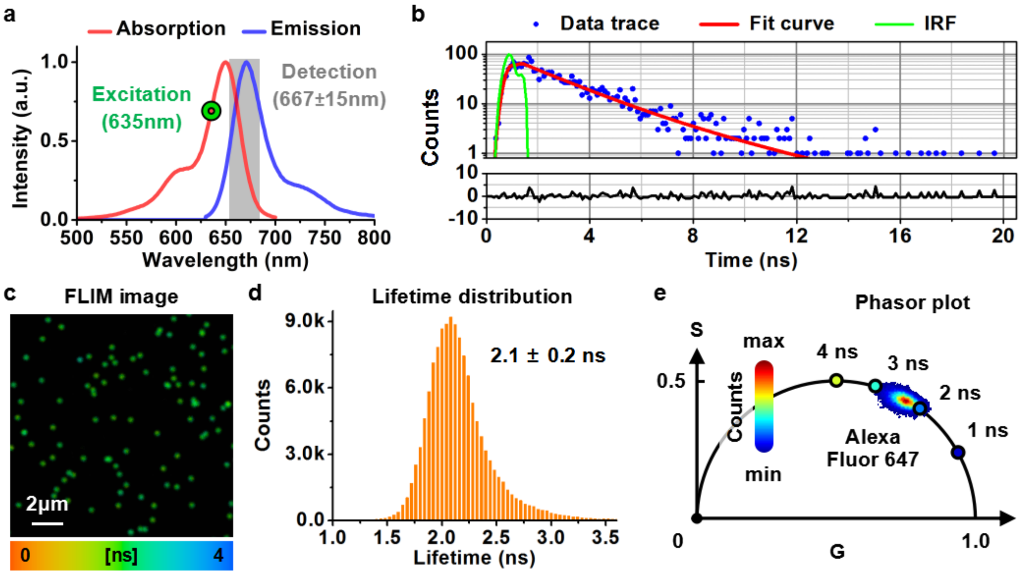

Figure S6 Parameter determination of the phasor algorithm by imaging a standard sample with a known lifetime in confocal mode. a Absorption and emission spectra of Alexa Fluor 647 and optical parameters of the setup. **b** Fitting analysis of a FLIM data by imaging a fluorescent beads sample labeled with Alexa Fluor 647. **c** FLIM image after the fitting procedure. **d** Lifetime distribution of all pixels. **e** Phasor plot of the FLIM image

For system calibration, we prepared a sample of 23 nm fluorescent beads labeled with Alexa Fluor 647.

The maximum absorption and emission wavelengths are 650 nm and 671 nm, respectively (**Figure S6**, **a**). The output signal from the sample was filtered using a bandpass filter (667 ± 15 nm) before entering the PMT detector. Figure S6b shows a fitting analysis of FLIM data, where the data trace (blue point), fit curve (red line), instrument response function (IRF) (green line), and residuals (black line) of a pixel in the image are presented. After the fitting procedure, the FLIM image was carried out (**Figure S6**, **c**) and the lifetime distribution showed a mean lifetime of 2.1 ns (**Figure S6**, **d**). The phasor plot of the FLIM image was obtained by phasor transformation (**Figure S6**, **e**). Finally, the data trace, fit curve, and lifetime value are used as criteria in our phasor algorithm.

To demonstrate the robustness of our method to variations in lifetime values, we included additional analyses by setting different lifetime values (**Figure S7**). The live HeLa cells were labeled with three dyes and analyzed using the confocal-FLIM mode. Following the fitting procedure, we obtained a FLIM image with a mean lifetime of 1.767 ns for the three dyes (**Figure S7**, **a**). Figure S7b depicts the fluorescence decay curve and lifetime distribution for all pixels in the image. The image contains a total of 8,848,718 photons, with a maximum of 64 photons per pixel. To compare, we used two random values (3.435 ns and 5.688 ns) in the phasor transformation, in addition to the real mean lifetime value obtained from the fitting procedure. It can be seen that the general integrity of the phasor plot remains unaffected by the use of varied lifetime values, but its center moves with the lifetime values (**Figure S7**, **c**). Following the phasor analysis, we obtained three 3-color confocal images (**Figure S7**, **d**) and calculated the intensity ratio of the three labeled structures in each image (shown in the insets in **Figure S7c**). Both images and intensity ratio produce good results across three different lifetime values, demonstrating our method's reliability despite potential errors in precise lifetime calibration.

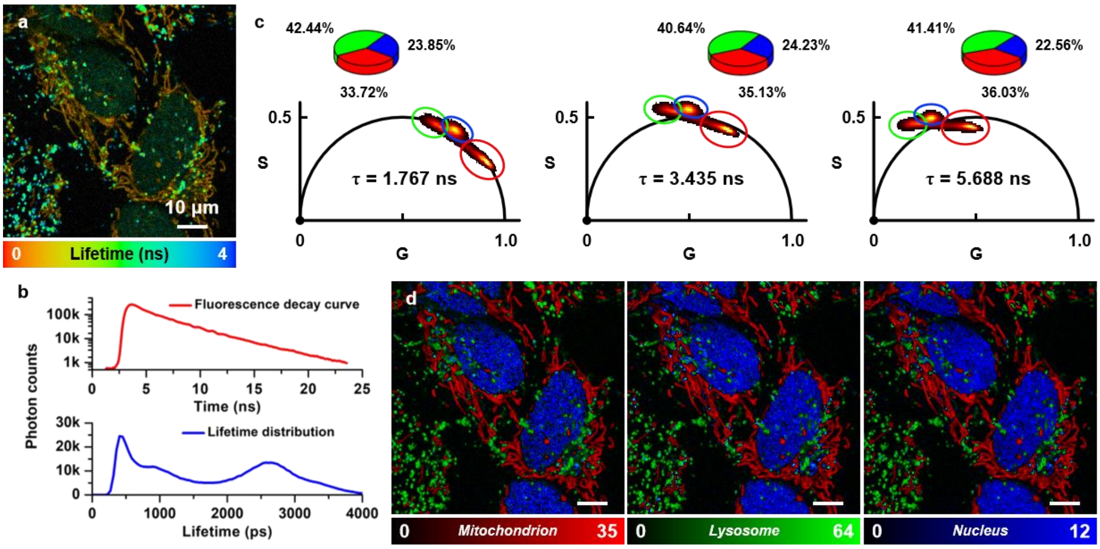

Figure S7 Phasor plots at different lifetime value settings. a FLIM image after fitting procedure (mean lifetime: 1.767 ns). **b** The fluorescence decay curve and the lifetime distribution. **c** Phasor plots at different lifetime values. **d** Three-color confocal images after phasor analysis. The color bars represent the number of photons. Scale bar, 10 μm

Phasor analysis and decomposition

By plotting raw FLIM data in the phasor plot, we can avoid some problems resulting from exponential analysis. A decay curve of fluorescence lifetime (**Figure S8**, **a**) is transformed in a point in complex phasor space (**Figure S8**, **b**) where, if the transformed curve is a single exponential, it will lie on the universal semicircle according to its lifetime. One important feature of phasor transformation that the

originates from vector algebra are the law of phasor addition. With two-exponential decays (**Figure S8**, **c**), the (green) phasor point (**Figure S8**, **d**) will lie along the line connecting the phasor position of the two contributing species, and its absolute position will depend on the relative photon contribution of the two species according to this property. For three components (**Figure S8**, **c**), the (magenta) phasor point (**Figure S8**, **d**) must be located inside the triangle set connecting three phasors of individual species. The same rules also apply and allow us to mathematically unmix more than three components, provided that they have similar spectra and separated fluorescence lifetimes.

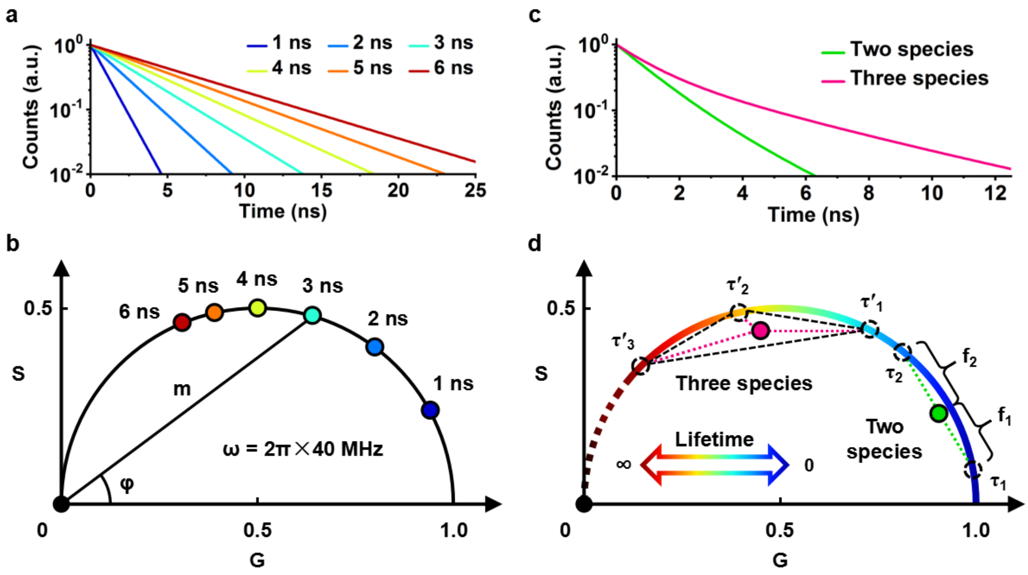

Figure S8 Phasor decomposition. **a** Simulation of single-exponential decays ranging from 1 ns to 6 ns and their representation in phasor space at a pulse rate of 40 MHz (**b**). **c** Simulation of multi-exponential decays for the combinations with two species (green) and three species (magenta) and their phasor transformation (**d**)

Determining the weight coefficient

In our FSTM system, the decay curve collected by the TCSPC module can record two complete processes of spontaneous emission for conventional probes whose lifetime is typically a few nanoseconds. According to the rules in the first two steps of phasor-FSTM, four images of intensities I_{g1}, I_{g2}, I_{d1}, and I_{d2} can be obtained from two temporal image stacks. Before unmixing, the intensity of images I_g and I_d is calculated as follows:

$$
\begin{aligned}
I_g &= \sum_{m,n}\sum_{t=t_g}^{t_d} I_g(x_m, y_n, t) = \sum_{m,n}\sum_{t=t_g}^{t_d}\left[I_{g_1}(x_m, y_n, t) + I_{g_2}(x_m, y_n, t)\right] \\
I_d &= \sum_{m,n}\sum_{t=t_d}^{T} I_d(x_m, y_n, t) = \sum_{m,n}\sum_{t=t_d}^{T}\left[I_{d_1}(x_m, y_n, t) + I_{d_2}(x_m, y_n, t)\right]
\end{aligned}
\qquad (6)
$$

where I_g and I_d are the intensities of the Gaussian and donut images, respectively; x_m, y_n, and t are the lateral and time coordinates; T is the pulse period; and t_g and t_d are the time coordinates of the Gaussian and donut laser pulses, respectively, relative to the reference signal ($t_g < t_d < T$).

Since the stochastic nature of spontaneous emission, which can be described by a single-exponential decay with fluorescence lifetime, it implies a fixed ratio δ between the photons in images I_g and I_d. Under the illumination of two lasers with the same wavelength and saturated stimulated absorption, the quantum yield of a fluorophore is the same. To achieve a balance between removing diffraction-limited

photons and preserving the desired photons emitted in the center of the Gaussian beam, we use the ratio of the photon number as a reference to determine the weight coefficient, i.e., $\delta = N_g / N_d$, where N_g and N_d are the total photon number (or mean intensity) of images I_g and I_d images, respectively. Therefore, the weight coefficient δ can be expressed as follows:

$$\delta = I_g / I_d = \sum_{m,n} \sum_{t=t_g}^{t_d} I_g(x_m, y_n, t) / \sum_{m,n} \sum_{t=t_g}^{t_d} I_d(x_m, y_n, t) \tag{7}$$

Consequently, the intensity of the final SRM image is $I_s(x, y) = I_g(x, y) - \delta I_d(x, y)$. The negative values that occur during image subtraction, which represent the complete suppression of the intensity signal, can be clipped to zero to ensure image quality [16]. In addition, applying low-pass filtering (smoothing) to the image I_d can produce an evenly distributed signal from sparse photons, helping to achieve sufficient and effective elimination in spatial modulation [17].

Collection and computational time

In our system, lifetime information is sampled in 256 time channels and the time for collecting a FLIM dataset (1024 × 1024 × 256) is a few seconds, which depends on the quantum yield of the fluorophores. On this basis, datasets are acquired with a dwell time of a few microseconds per pixel. The execution time for transforming a FLIM dataset to the phasor plot is on the order of seconds to minutes, which can further reduce by exploiting Graphics Processing Unit (GPU) acceleration. The performances for smaller datasets with the current implementation scale linearly with the number of pixels in the image. However, the FLIM datasets are typically tens to hundreds of MB in size (depending on the pixels of the FLIM image and the number of photons collected) and RAM saturation can heavily slow down the processing time. This issue should be addressed on a case-by-case basis according to the performance of the computer.

Calculating the laser power density

The laser power density (G) is calculated according to the formula $G = 4P/(\pi D^2)$, where P is the laser power, and D is the diameter of the laser beam defined by the position where the intensity is reduced to $1/e^2$ of the peak. The laser power is measured at the back aperture of the objective with a power meter (PM100D&S130C, Thorlabs, Newton, NJ, USA). For a Gaussian (TEM_{00} mode) laser, the diameter of the laser beam has a linear relationship with the full width at half maximum (FWHM); i.e., $D \approx 1.7 \times$ FWHM (**Figure S9**, **a**). The PSFs of the Gaussian and donut lasers are measured by imaging Au nanoparticles with sizes below the diffraction limit (150 nm). After calibrating the system, two focal spots are highly overlapped in space, which lays the foundation for acquiring high-quality phasor-FSTM images (**Figure S9**, **b**). For the Gaussian spot, the intensity profile is Gaussian-fitted, yielding an FWHM value of 240.6 nm and therefore a diameter of 409.0 nm (**Figure S9**, **c**). For the donut laser spot, the intensity profile is fitted using two Gaussian curves, yielding FWHM values of 166.1 nm and 164.0 nm (**Figure S9**, **d**). The sum of the two diameters is taken as the diameter of the donut laser spot; i.e., $D_d = D_{d1} + D_{d2} = 1.7 \times (FWHM_{d1} + FWHM_{d2}) = 561.2$ nm. In the experiments, the power density is calculated to be 4.41 kW cm^{-2} for 5.8 μW Gaussian excitation and 2.95 kW cm^{-2} for 7.3 μW donut excitation. It can be seen that the power density of the donut laser in the focal plane is only about two-thirds that of the Gaussian laser. Therefore, when the wavefront shape of the excitation light is ignored, the proposed scheme in this paper can be regarded as a sort of confocal imaging with an excitation frequency of 80 MHz.

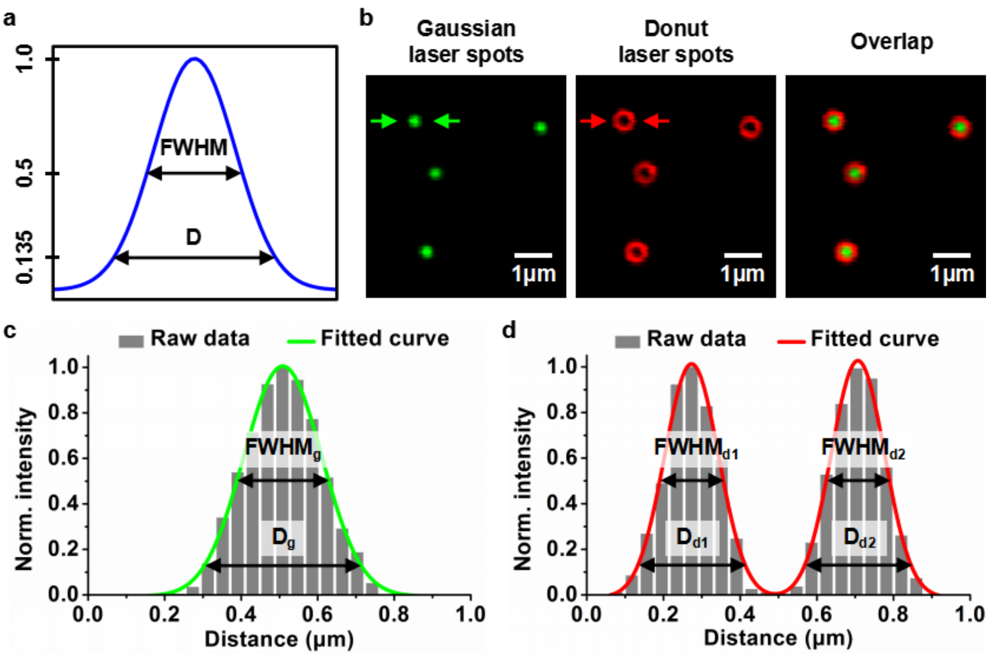

Figure S9 Calculating the diameter of the laser focal spots. a The FWHM and the diameter (D) of a Gaussian beam. **b** Scattering images of 150 nm Au nanoparticles illuminated by Gaussian and donut laser spots, as well as their overlapping image. **c** Normalized intensity profile of the Gaussian laser spot marked by green arrows in **b**. **d** Normalized intensity profile of the donut laser spot marked by red arrows in **b**

Phasor-FSTM for Two-Color Super-Resolution Imaging of Fluorescent Beads

We ordered a bead sample from GATTAquant (www.gattaquant.com), which was prepared using a mixture of two kinds of fluorescent beads labeled with STAR 635P and Alexa Fluor 647, both 23 nm in diameter. FLIM imaging was carried out under the illumination of two excitation lasers, and the FLIM image was obtained after fitting procedure (**Figure S10**, **a**). The distribution histogram shows a mean lifetime of 2.2 ± 0.2 ns (**Figure S10**, **b**). The whole fluorescence decay curve (red line) after activating binning of all pixels inside the image was divided into two parts by the donut pulse in a laser pulse period (**Figure S10**, **c**). The time delay was determined to be 10.6 ns by calculating the interval between the peaks of two spontaneous emission processes. By treating as the boundary the channel where the peak of the donut pulse is located, a FLIM file (.sdt) is split into two datasets (temporal image stacks) using our phasor algorithm. After phasor transformation, the phasor plots of two image stacks were obtained (**Figure S10**, **d**). Photon extraction in two phasor plots was performed to obtain four images of intensities I_{g1}, I_{g2}, I_{d1}, and I_{d2}, and two super-resolution images (phasor-FSTM images) of intensities I_{s1} and I_{s2} were presented at a weighted coefficient of 2 (**Figure S10**, **e**). The selection regions (blue and magenta circles) were plotted according to the relative lifetime and concentration of two fluorophores. Finally, we realized two-color super-resolution imaging in the fluorescent beads sample (**Figure S10**, **f**). The intensity profiles in the two-color SRM image were measured, and two adjacent fluorescent beads show FWHMs of 87.3 nm and 91.6 nm (**Figure S10**, **g**). We further applied decorrelation analysis (DCA) for quantitative resolution measurement, which is based on an individual image without further requirements or a priori knowledge [18]. With this method, the resolution was expressed as $2/k_c$, where k_c is the cutoff frequency. A precision value of 90.2 nm (k_c = 0.368) for the two-color SRM image was obtained using DCA (**Figure S10**, **h**), which is one-seventh of the excitation wavelength.

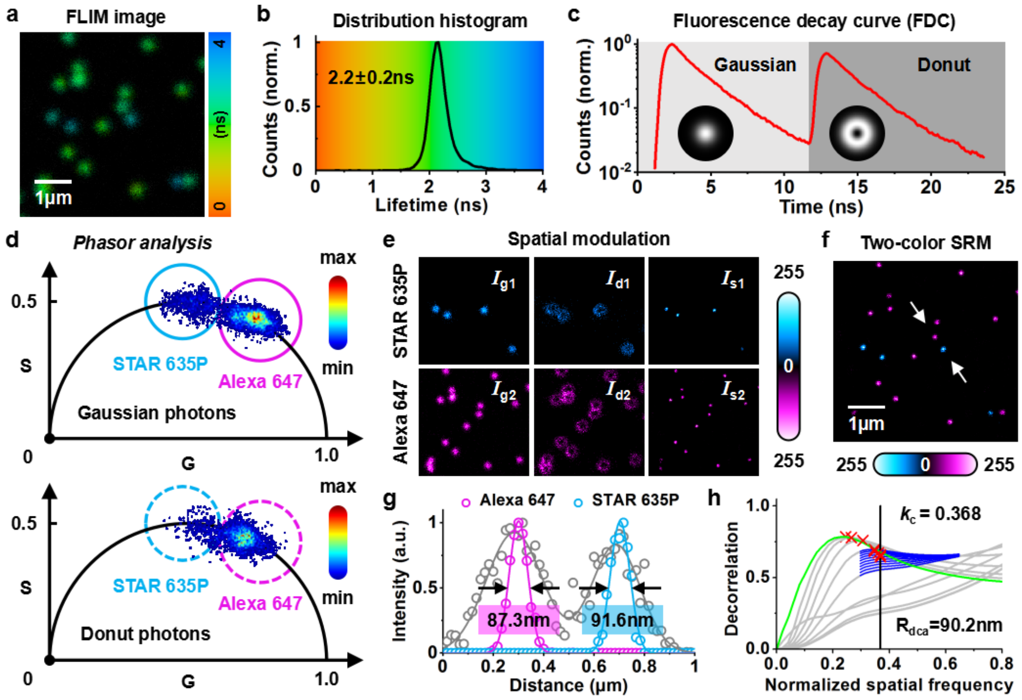

Figure S10 Phasor-FSTM for two-color super-resolution imaging of a fluorescent beads sample. a FLIM image after the fitting procedure. **b** Distribution histogram. **c** Fluorescence decay curve. **d** Photon extraction in phasor space. **e** Spatial modulation. **f** Two-color SRM image. **g** Normalized intensity profiles across the beads marked by the white arrows in **f**. **h** Resolution estimation by decorrelation analysis (pixel size: 16.6 nm)

Deconvolution

Deconvolution is used in our work to reduce the blurring effect caused by non-uniformly distributed signals of sparse photon, thereby further enhancing image contrast and resolution. We have carefully selected the Huygens software (Scientific Volume Imaging, SVI) for deconvolution, adhering to its default parameter settings to standardize our approach across all datasets. Detailed parameters of the deconvolution process are as follows: 1) background: automatic estimation; 2) estimate mode: lowest; 3) area radius (micron): 0.7; 4) deconvolution algorithm: CMLE; 5) maximum iterations: 15; 6) signal-to-noise ratio: 7; 7) quality threshold: 2; 8) iteration mode: optimized; 9) brick layout: auto; and 10) PSFs per brick: auto.

We also demonstrated the efficacy of our deconvolution strategy by providing a comparative analysis of confocal and FSTM images of fluorescent beads, both pre- and post-deconvolution (**Figure S11**, **a**). Two beads marked by white arrows in four images have FWHMs of 274/249 nm, 164/154 nm, 117/114 nm, and 83/81 nm obtained by Gaussian fitting, respectively (**Figure S11**, **b**). Also, their SBRs are 12.50, 16.76, 19.05, and 23.61, respectively (**Figure S11**, **c**). This comparison demonstrates a further improvement in resolution following deconvolution, as evidenced by lower FWHMs and higher SBRs in the deconvolved images. Such enhancements validate our deconvolution approach and ensure that the final phasor-FSTM images have higher resolution and quality. In the raw data in Figure S11, the total number of photons before channel separation is 2,246,821, while it is 1,271,127 and 975,699 in the Gaussian and donut channels, respectively. Furthermore, the maximum number of photons in pixels before channel separation is 48, while the maximum number of photons in Gaussian and donut images obtained after channel separation is 39 and 22, respectively. The detailed photon counts highlight our method's ability to produce high-quality super-resolution images with optimized photon usage,

adhering to the principle that live-cell imaging requires low-intensity illumination without sacrificing image quality.

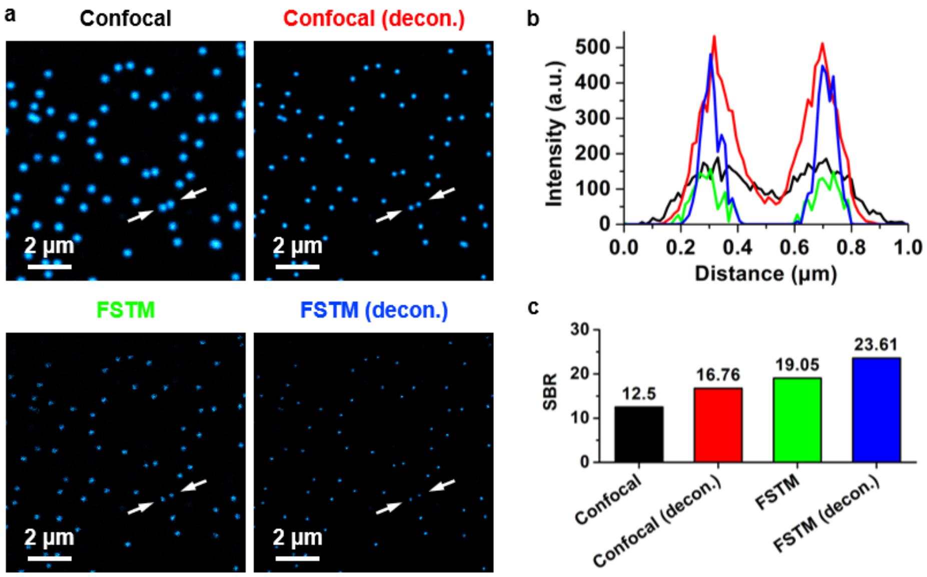

Figure S11 Comparison of confocal and FSTM images before and after deconvolution. a Confocal and FSTM images before and after deconvolution. FOV: 11 × 11 μm^2; pixel size: 800 × 800 pixels; **b** The intensity profiles of two fluorescent beads marked by write arrows. **c** The SBRs of four images

Morphological analysis

For multiplexed analysis of the state/function and morphology of organelles, we used the open-source image analysis platform ImageJ/Fiji [19,20]. Two plug-ins were inserted into the ImageJ/Fiji shareware: the Mitochondria Analyzer and the Mitochondrial Network Analysis (MiNA) toolset. The Mitochondria Analyzer plugin is publicly available and includes a graphical user interface to facilitate preprocessing, parameter optimization, image thresholding, and automated morphofunctional analysis of images or image stacks [21]. The parameters were as follows: 1) Rolling (micron): 1.25; 2) Radius: 3.50; 3) Max Slope : 1.8; 4) Gamma: 0.8; 5) Outlier Radius: 4.1 pixels; 6) Threshold Method: Weighted Mean; 7) Block Size: 1.250 microns; 8) C-Value: 10. We used the Mitochondria Analyzer to measure the areas of subcellular structures in Fig. 2. Mitochondria and lysosomes in confocal and FSTM images were identified by adaptive thresholding (**Figure S12**, **a**), and their perimeter and aspect ratio (AR) were measured (**Figure S12**, **b**). In confocal and FSTM, the total perimeter of the mitochondria/lysosomes is 401.35/407.80 μm and 278.68/256.28 μm, and the mean ARs are 2.45/1.83 and 2.33/1.92, respectively. In the comparison of confocal and FSTM, the total/mean perimeter of the subcellular structures was reduced without much change in AR. This demonstrates that the proposed method not only effectively improves the spatial resolution but also causes no obvious change to the sample structure.

As a relatively simple pair of macros making use of existing ImageJ plug-ins, MiNA incorporates optional preprocessing steps to enhance the quality of images before converting the images to binary and producing a morphological skeleton for calculating nine parameters that quantitatively capture the morphology of the mitochondrial network. This allows for semi-automated analysis of mitochondrial networks in cultured mammalian cells [22]. Mitochondria exist in a dynamic cycle of fusion and fission whose balance directly influences the morphology of the mitochondrial network. As such, it is necessary to conduct morphological analysis of mitochondria. MiNA recognizes only two distinct

object types of mitochondrial structures in a skeletonized image: networks (structures with at least one junction) and individuals (no junctions). All pixels within a skeleton are grouped into three categories: end-point pixels, slab pixels, and junction pixels. We used MiNA to analyze mitochondria in confocal and FSTM images to obtain the corresponding outline and skeletonized images, in which 311/254 individuals and 52/15 networks were contained in the skeletonized image (**Figure S12**, **c**). More details can be seen in the outline image of the FSTM, including the mitochondrial cristae. Compared with the confocal skeletonized image, the mitochondrial network skeleton in the corresponding FSTM image is uncluttered and more precise. The parameters were as follows: 1) Blocksize: 127; 2) Histogram Bins: 256; 3) Maximum Slope: 3; 4) Radius: 2; 5) Mask Strength: 0.60.

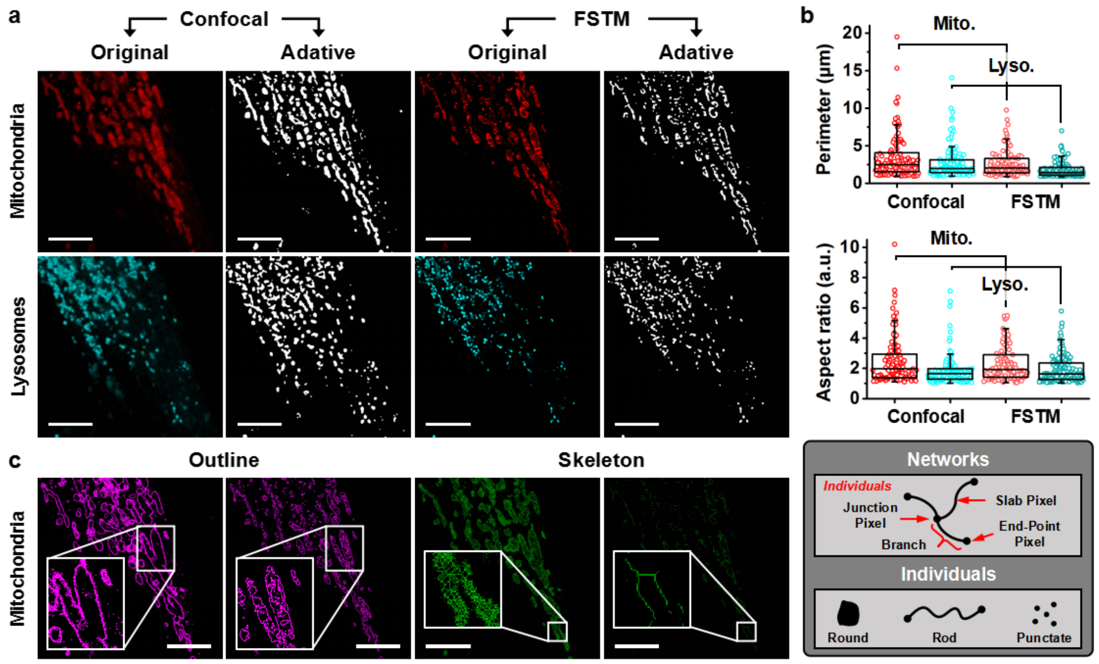

Figure S12 Morphological analysis. a Object identification using adaptive thresholding on the confocal and FSTM images of mitochondria and lysosomes. **b** Quantitative comparison of the perimeter and aspect ratio between confocal and FSTM images. **c** The outline and the skeletonized images of mitochondria by MiNA. Inset: schematic diagram of mitochondrial network and individuals. Networks are mitochondrial structures with at least a single node and three branches. Individuals include a punctate (a single pixel in the skeletonized image), rods (unbranched structures with two or more pixels in the skeletonized image), and large/round structures (which are reduced to rods, or occasionally small networks, during skeletonization). Scale bars, 5 μm (a, c)

Calculating the SBR

The SBR is defined as the ratio of the average intensity value of pixels in the signal area (in the image) to that of pixels in the background area [23]. A global threshold (φ) that can be used to convert an intensity image to a binary image is computed using Otsu's method, where φ is a normalized intensity value that lies in the range [0, 1]. Otsu's method chooses the threshold to minimize the intraclass variance of the thresholded black and white pixels. The pixels in the signal area do not contain the background value, and the pixels in the background area do not contain the value of the target signal. Therefore, the SBR of the images in this paper is given by

$$SBR = 10\left|\lg(\mu_s / \mu_b)\right| \tag{8}$$

where μ_s is the mean pixel value within the image identified as a structure of interest or "signal", and μ_b is the mean pixel value within the image of equal size identified as containing no structures of interest or "background".

Labeling Specificity and Lifetime of the Probes

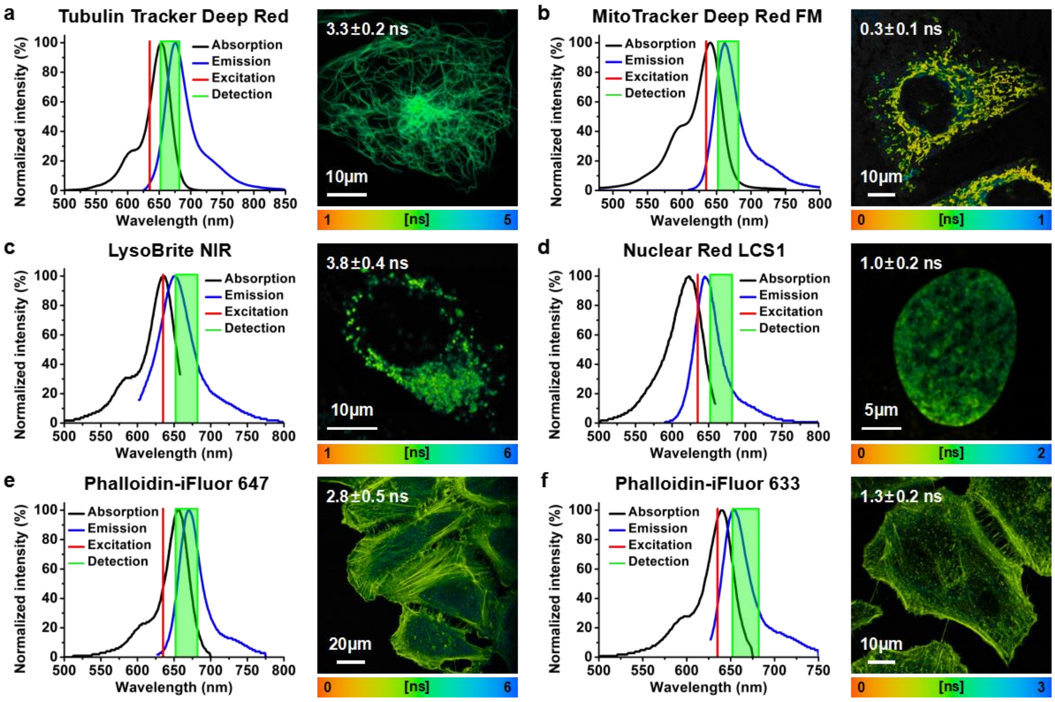

Figure S13 Absorption and emission spectra of the probes and the FLIM images after labeling the structure in confocal imaging mode. a Tubulin Tracker Deep Red. **b** MitoTracker Deep Red FM. **c** LysoBrite NIR. **d** Nuclear Red LCS1. **e** Phalloidin-iFluor 647. **f** Phalloidin-iFluor 633

The probes used in this paper are all commercially available, so their labeling specificity has been validated [24–30]. We used several staining kits based on the parameters of the experimental system and performed FLIM imaging in confocal mode after labeling the structure (Invitrogen™ Tubulin Tracker Deep Red and MitoTracker Deep Red FM, ThermoFisher Scientific; LysoBrite™ NIR, Nuclear Red™ LCS1, Phalloidin-iFluor® 647 Conjugate and 633 Conjugate, AAT Bioquest). Tubulin Tracker™ Deep Red is a conjugate of Docetaxel and a photostable deep-red fluorophore that easily enters the cells and specifically labels polymerized tubulin, which can be used in a wide variety of cell types. Compared to the existing live cell Tubulin labeling methods, it provides uniform staining, superior photobleach resistance, and no measurable cytotoxic effects, even after 24-hour incubation. MitoTracker™ Deep Red is a cationic carbocyanine that has been used in scientific research for more than a decade. LysoBrite™ dyes can stay in live cells for more than a week with very minimal cell toxicity and survive a few generations of cell division. They significantly outperform the equivalent LysoTracker™ dyes (from Invitrogen). LysoBrite™ NIR, the proprietary lysotropic dye used in the kit, selectively accumulates in lysosomes, probably via the lysosome pH gradient. Nuclear Red™ LCS1 is a fluorogenic, DNA-selective and cell-permeant dye for analyzing DNA content in living cells. It has red fluorescence that is significantly enhanced upon binding to DNA. Phalloidin-iFluor® 647 and 633 conjugate (equivalent to Alexa Fluor® 647- or 633-labeled phalloidin) selectively binds to F-actins. Used at nanomolar concentrations, phalloidin derivatives are convenient probes for labeling,

identifying, and quantitating F-actins in formaldehyde-fixed and permeabilized tissue sections, cell cultures, or cell-free experiments. Figure S13 gives the absorption and emission spectra of those probes, showing that they allow for simultaneous excitation and detection under the same system configuration. After the fitting procedure, FLIM images and the mean lifetimes of the probes were obtained. Their different lifetimes allow multicolor super-resolution imaging in phasor-FSTM. Based on the advantages of the method, a wide variety of probes can be candidates for multicolor SRM imaging studies, including organic dyes, quantum dots (QDs), lanthanide-doped up-conversion nanoparticles (UCNPs), and carbon dots (CDs).

Multicolor imaging

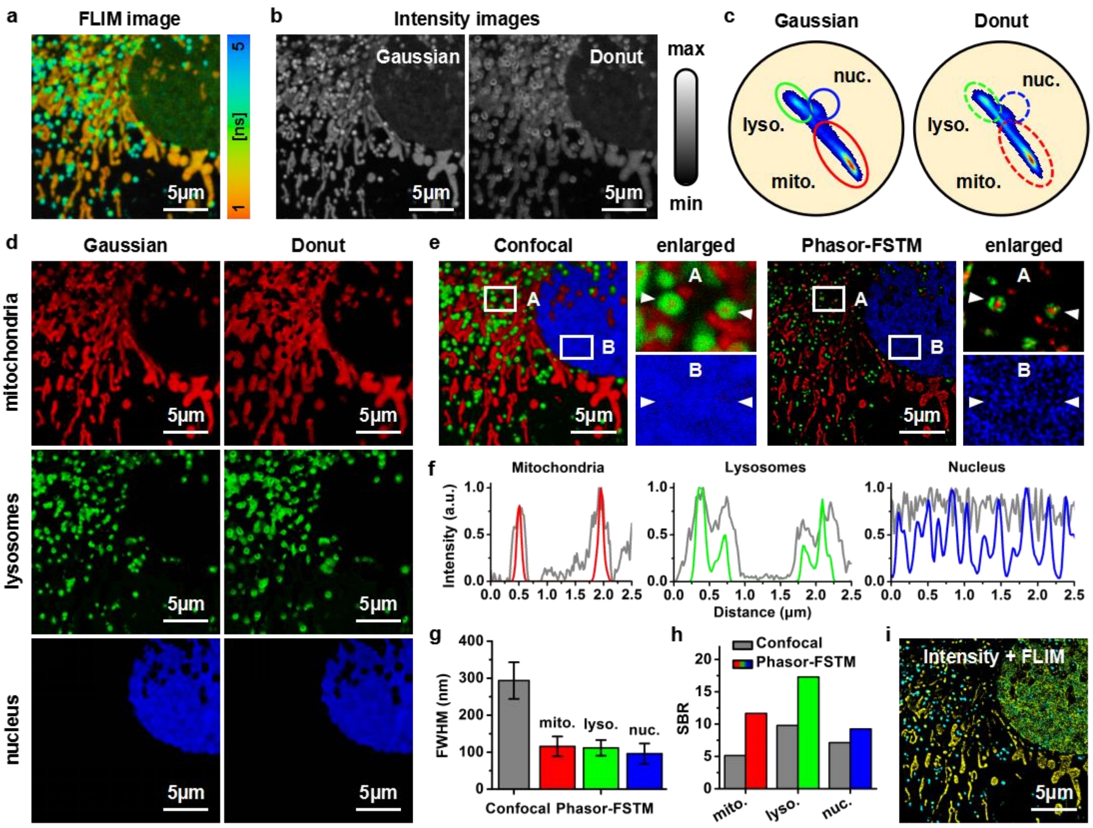

Figure S14 Phasor-FSTM for three-color super-resolution imaging. a A local FLIM image of a single HeLa cell. **b** Gaussian and donut intensity images obtained by temporally modulation. **c** Phasor plots and photon extraction. **d** Intensity images for each of the three labeled structures consisting of Gaussian and donut photons. **e** Comparison of three-color confocal and phasor-FSTM images. **f** Normalized intensity profiles along the lines marked by white triangles in **e**. **g** FWHM values of the intensity profiles across the structures. **h** Comparison of the SBR between the confocal and phasor-FSTM images. **i** Combined image. Brightness represents the number of photons, and color represents the fluorescence lifetime

Using three kits (MitoTracker Deep Red FM, LysoBrite NIR, and Nuclear Red LCS1), we performed three-color super-resolution imaging in phasor-FSTM (**Figure S14**). Figure S14a shows a localized FLIM image of a single HeLa cell. The fluorescence lifetimes obtained by the fitting procedure were transferred into a color scale. By summing up the photon numbers in the Gaussian and donut time channels, two intensity images were obtained (**Figure S14**, **b**). The phasor plots of two image stacks were carried out after the phasor transformation, showing three separate regions of photon distribution

(**Figure S14**, **c**). The photons were extracted to form six images, where the ellipses in red, green, and blue locate the fluorescence signal of mitochondria, lysosomes, and the nucleus, respectively (**Figure S14**, **d**). Figure S14e compares the three-color confocal and phasor-FSTM images, having higher resolution in phasor-FSTM than that in confocal. The effect was also clearly visible in the normalized intensity profiles (**Figure S14**, **f**), which are marked by white arrows in Fig. S14e. The excellent lifetime separation afforded by the dyes allowed us to achieve low crosstalk between labeled structures. The mean FWHMs of 0.182λ, 0.176λ, and 0.151λ were obtained in the phasor-FSTM images for the structures of the mitochondrion, lysosome, and nucleus (**Figure S14**, **g**) and the images feature higher SBR in phasor-FSTM than that in confocal (**Figure S14**, **h**). By combining the intensity of the phasor-FSTM image shown in Fig. S14e and the color of the image shown in Fig. S14a, the intensity and lifetime information from the sample can be presented simultaneously (**Figure S14**, **i**). Although the absolute value of lifetimes has changed in the combined image, the lifetime difference on labeled structures still exists, and is able to provide additional information on the basis of the intensity image.

Without the implementation of spatiotemporal modulation, multicolor confocal imaging can be realized using only phasor analysis. In confocal imaging mode, we collected a FLIM dataset and obtained an intensity image by summing up the photon numbers in all time channels (**Figure S15**, **a**). The phasor plot was carried out after the phasor transformation, showing four separate regions of photon distribution (**Figure S15**, **b**). The photons were extracted to form four color-coded intensity images, where the ellipses in yellow, magenta, green, and cyan locate the fluorescence signal of mitochondria, lysosomes, the microtubule, and the nucleus, respectively (**Figure S15**, **c**). Figure S15d shows the FLIM image after the fitting procedure. The four-color confocal image is obtained by superimposing the images in Fig. S15c to show the spatial distribution of the four subcellular structures in a single cell (**Figure S15**, **e**).

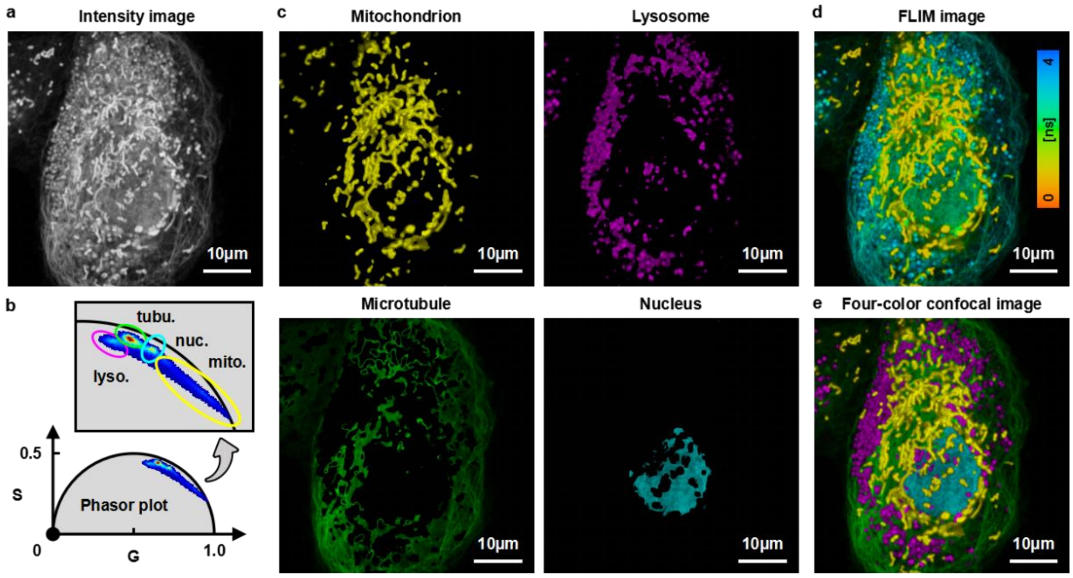

Figure S15 Four-color confocal imaging using phasor analysis. a Intensity image of a single HeLa cell consisting of lysosome, mitochondrion, microtubule, and nucleus structures. **b** Phasor plots and photon extraction. **c** Intensity images for each of the four labeled structures. **d** FLIM image of a single HeLa cell. **e** Four-color confocal image after using phasor analysis

Photobleaching Resistance

Although the method uses two laser beams for excitation, the time delay between them is much longer than the fluorescent lifetime of the dyes, ensuring that there is no re-excitation during the spontaneous emission of fluorescence. We prepared a cell sample where lysosomes were labeled with a commercial staining kit as before, and measured the variation of fluorescence intensity over the scanning time (**Figure S16**, **a**). In phasor-FSTM imaging mode, the image still maintained a high fluorescence intensity with only 23% attenuation after continuous illumination for 400 s in a field of view (FOV) of 15 × 15 μm^2 (**Figure S16**, **b**). Importantly, the SBR of the image remains almost unchanged during long-term imaging, which is crucial for the study of microstructures and their interactions at the nanoscale (**Figure S16**, **c**). In addition, we present a comparison between phasor-FSTM and a confocal scheme (pulse rate: 80 MHz) in terms of photobleaching resistance (**Figure S16**, **d**). At the same laser power density (7.6 kW/cm^2 for the Gaussian beam in confocal mode, and 4.1 kW/cm^2 for the Gaussian beam and 3.5 kW/cm^2 for the donut beam in phasor-FSTM), imaging was performed continuously for 20 min (1046 frames) at several different locations of the sample. Compared to confocal, the decay rate of the intensity in phasor-FSTM was even lower over 20 min. The reason for this is that although the total power density of the two schemes is the same, the average power density of the phasor-FSTM method is lower. In this respect, phasor-FSTM is equivalent to a conventional confocal system, but with higher spatial resolution and strong structural identification ability.

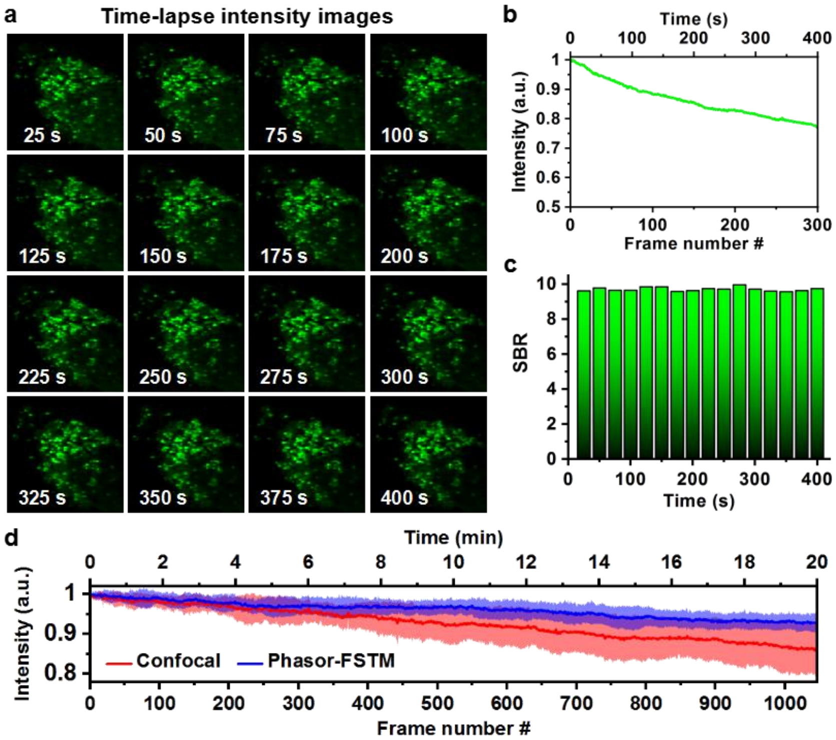

Figure S16 Test and comparison of photobleaching resistance. a Time-lapse intensity images of lysosomes in a live cell in phasor-FSTM imaging mode. FOV: 15 × 15 μm^2. **b** Variation of fluorescence intensity over the scanning time. **c** SBRs of the images in **a**. **d** Comparison of fluorescence intensity attenuation between phasor-FSTM and confocal imaging modes. FOV: 140 × 140 μm^2; pixel size: 512 × 512 pixels; pixel dwell time: 4.37 μs

A variety of fluorescent dyes from suppliers such as ThermoFisher Scientific, AAT Bioquest, Abberior, SpiroChrome, and Genvivo are available for labeling living cells. However, in our paper, we only tested and used a small subset that met our system's needs. The optimal excitation wavelength of the dyes used for four-color imaging vary, but all are near 635 nm (**Figure S17**, left). LysoBrite NIR,

MitoTracker, and Nuclear Red LCS1 have an excitation efficiency greater than 80 % at 635 nm, while TubulinTracker has an excitation efficiency of only 58% and works best with light at 653 nm (**Figure S17**, right). To compensate for TubulinTracker's lower efficiency, we used it at ten times the concentration of the other dyes.

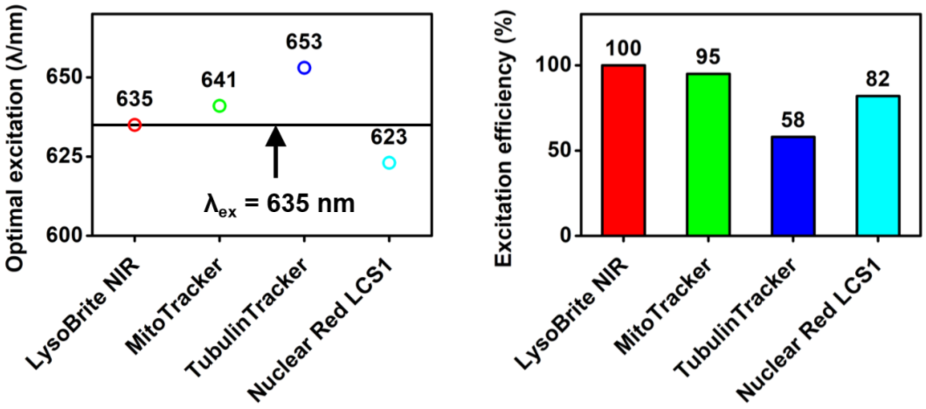

Figure S17 The optimal excitation wavelength of dyes used in four-color super-resolution imaging (left), and their excitation efficiency at 635 nm excitation wavelength (right)

Another issue needs to be taken into account. Because the quantum yields of dyes are different, they may have different photobleaching rates under the same scheme of laser illumination. Therefore, we performed a series of long-term imaging of subcellular structures labeled by each of the four staining kits in confocal mode to test their photobleaching resistance. HeLa cell samples were labeled and imaged in confocal imaging mode. Figure S18 shows the changes in fluorescence intensity of four commercial staining kits at laser power of 1.25 μW. After continuous imaging for up to 22 min, the intensity curves exhibited different photobleaching resistance. Unlike the spectral separation method, which uses multiple detectors to capture different emission bands, our method uses a single PMT detector to collect photons from all of the labeled dyes. As a result, when the same settings, such as excitation wavelength and power, and detection efficiency, are used, the brightness of the fluorescence from these dyes should be similar. This also influences the length of the effective time in multicolor imaging. As a result, the dyes used must be carefully chosen. Our method can overcome some of the challenges that existing multicolor SRM techniques face by selecting and optimizing fluorophores appropriately, allowing for long-term, multicolor super-resolution imaging of living cells and other biological samples.

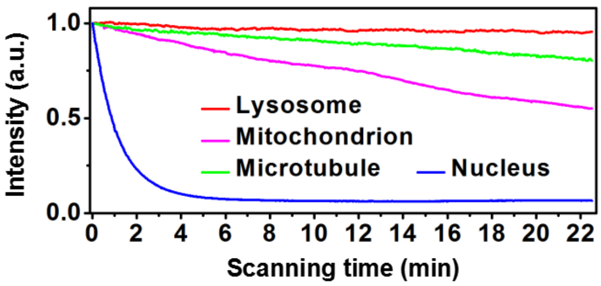

Figure S18 Changes in fluorescence intensity of four commercial staining kits in confocal mode. Lysosomes (red, LysoBrite NIR), mitochondria (magenta, MitoTracker Deep Red FM), microtubules (green, Tubulin Tracker Deep Red), and the nucleus (blue, Nuclear Red LCS1). Laser power: 1.25 μW; FOV: 54 × 54 μm^2; pixel size: 1024 × 1024 pixels; pixel dwell time: 2.58 μs

Time-lapse imaging

The time-lapse phasor-FSTM images from the red rectangle in Fig. 5b were time-color coded and stacked, and the images of mitochondria and lysosomes were separated for comparison (**Figure S19**, **a**). It can be seen that the mitochondria undergo morphological changes but do not move appreciably, as do the lysosomes that are attached to them. Most strikingly, one of the lysosomes travels through the mitochondria following the motion trail (white dashed line), interacting with one mitochondrion as it moves. The lysosome moves rapidly when it is not in contact with the mitochondria, but slows down when it gets closer to them. The total movement distance reaches ~9 μm during the imaging time of 318 s, and a maximum speed of 0.257 μm/s appeared between the 11th and the 12th frames (**Figure S19**, **b**). The result is consistent with the literature [31].

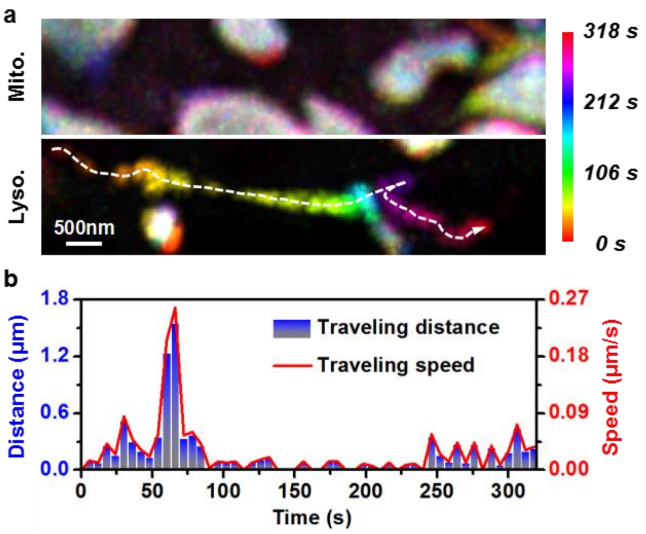

Figure S19 Dynamics of mitochondria and lysosomes of Figure 5d. a Time-color-coded phasor-FSTM stack images of mitochondria and lysosomes (different colors indicate different imaging times). The white dotted line shows the motion trail of the lysosome. **b** Traveling distance (blue) and speed (red) of a lysosome in **a**

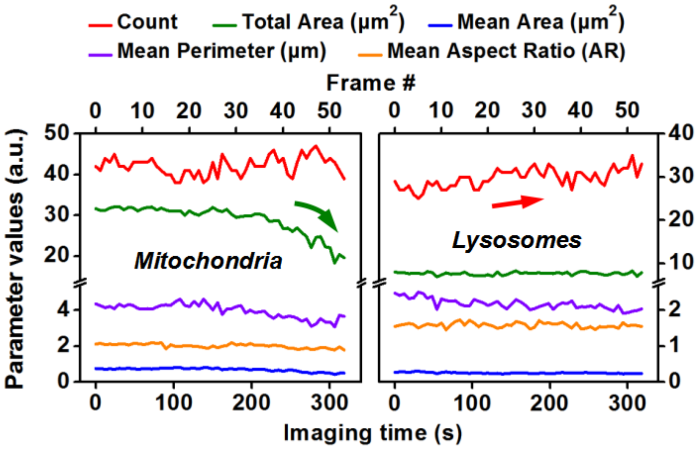

Figure S20 2D morphological analysis of mitochondria and lysosomes. The count, total area, mean area, mean perimeter, and mean aspect ratio (AR) curves of mitochondria and lysosomes in full-field phasor-FSTM images over the imaging time. The area and perimeter characterize the size of the structure, while the structure shape is defined by the form factor (FF) and aspect ratio (AR)

We performed 2D morphological analysis of mitochondria and lysosomes on full-field phasor-FSTM images, in which the count, total area, mean area, mean perimeter, and mean aspect ratio (AR) curves of mitochondria and lysosomes were shown to change over time. During the imaging time of 318 s (54 frames), the count and the mean AR of mitochondria do not significantly change, while the total/mean area and mean perimeter decreased gradually at the later stage of imaging. In contrast, the total area and the mean AR of lysosomes remained constant, but the count went up, leading to a decrease in the mean area and mean perimeter. The results show that the shrinkage of the mitochondrial structure is accompanied by an increase in the number of lysosomes, promoting the process of mitochondrial autophagy. Despite no change to the total lysosomal area, some external factors increased the number of lysosomes and reduced their average size (mean area and mean perimeter), suggesting increased mitochondrial dysfunction.

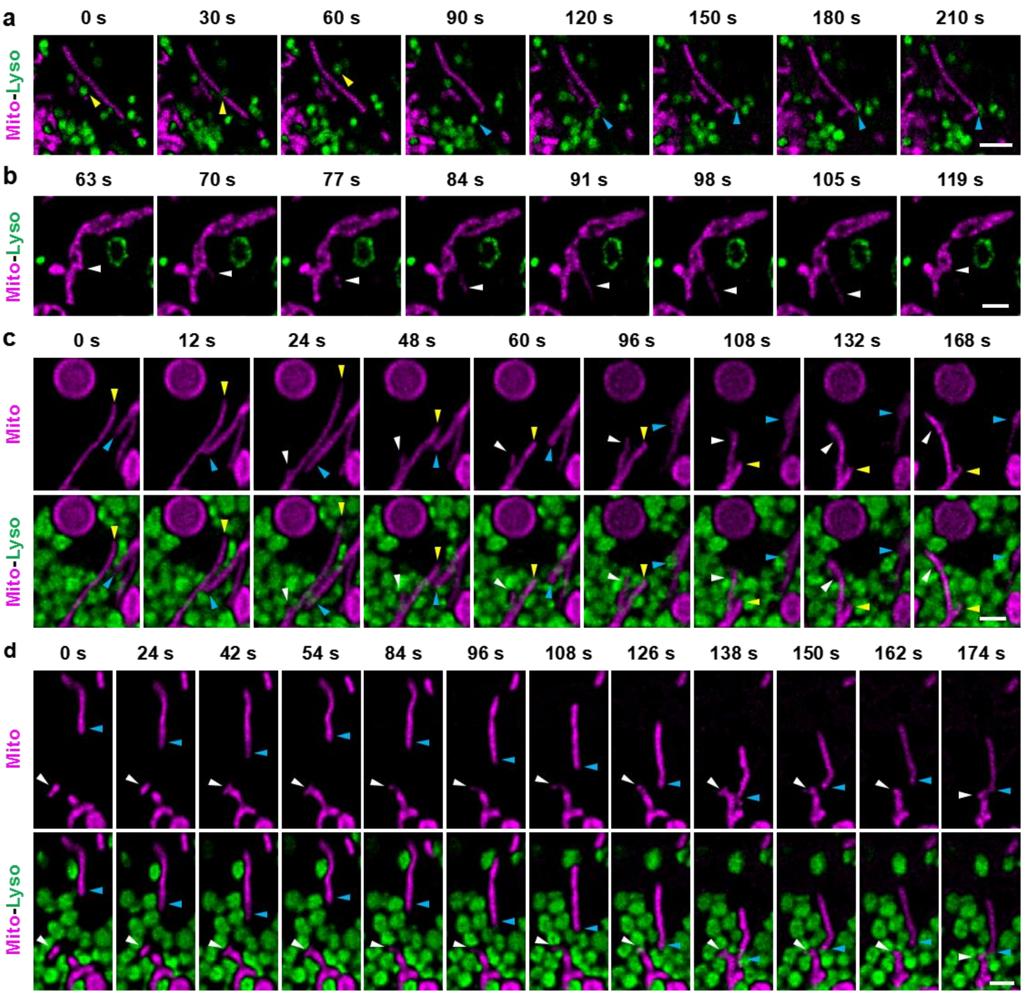

Figure S21 Two-color phasor-FSTM images of lysosomes and mitochondria in live cells. a-d Representative time-lapse phasor-FSTM super-resolution images reveal different dynamic physical interactions between lysosomes and mitochondria in live HeLa cells stained with LysoBrite NIR (green) and MitoTracker Deep Red FM (magenta). More frames are shown in Supplementary Videos 3-7. Scale bars, 2 μm (a) and 1 μm (b-d)

We performed a series of experiments for long-term two-color phasor-FSTM imaging, and observed abundant dynamic interactions between mitochondria and lysosomes (**Figure S21**, and **Supplementary Videos 2–7**). Mitochondria and lysosomes can directly interact via degradative and nondegradative processes in healthy mammalian cells. Figure S21a show two lysosomes successively contact tethering

and untethering with a long and slender mitochondrion. The processes of contact tethering and untethering involve mitochondrion–lysosome contact sites, which may mediate additional functions, such as the inter-organelle transfer of metabolites, including lipids, calcium, or iron [32]. Dynamic mitochondrial tubulation can maintain mitochondrial DNA integrity and interchanging mitochondrial material. These tubules sometimes retract back to the original mitochondrion (**Figure S21**, **b**; 5 s/frame, with 2 s dark recovery), and sometimes separate from the original (**Figure S23**, **c**). In addition, the tubule, guided by lysosomes, can build a bridge between the original mitochondrion and an adjacent one, allowing them to fuse. In Fig. S21c, a mitochondrion (marked by the yellow arrow) retracts and grows from another site (marked by the white arrow). It also makes contact with another mitochondria (marked by the blue arrow) during this process. Alternatively, the mitochondria moved a few microns, surrounded by lysosomes, and then fused with the mitochondrion at the bottom of the image (**Figure S21**, **d**).

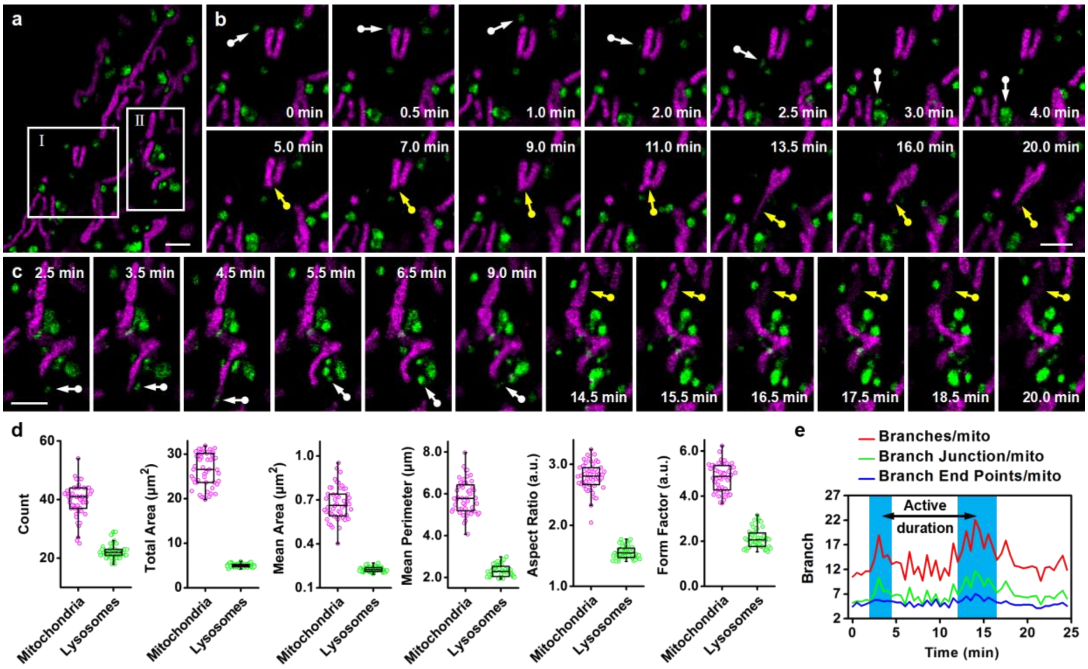

Figure S22 Phasor-FSTM monitors the continuous dynamic processes of mitochondria and lysosomes. a A two-color phasor-FSTM image of mitochondria and lysosomes in a large field of view. **b**-**c** Multiple dynamic processes of mitochondria and lysosomes under continuous imaging in two white rectangular labeled regions in **a**. **d** 2D morphological analysis of mitochondria and lysosomes. Data are presented as the means ± SE. **e** The curves of branches, branch junction, and branch end points per mitochondrion in full-field phasor-FSTM images over the imaging time. Blue areas indicate the "active duration" of the mitochondria. Scale bars, 2 μm (a-c)

In long-term phasor-FSTM imaging over a time course of 24.5 min, we observed multiple continuous dynamic processes of mitochondria and lysosomes in the same FOV (**Figure S22**, **a**). We focus on two regions, marked by white rectangles in Fig. S22a, where the mitochondria and lysosomes are concentrated. A lysosome (marked by the white arrow) contact tethered and untethered twice with a mitochondrion within 3 min (each contact lasted less than 30 s) and then rapidly fused with a larger lysosome located at the bottom of the image (**Figure S22**, **b**). After a few minutes, the mitochondrion (marked by the yellow arrow) that the lysosome had previously touched began to fuse with the neighboring one, and then the fused mitochondria underwent tubulation. In the other case, some lysosomes attached to several connected mitochondria (**Figure S22**, **c**). We can see that a lysosome

moved over to the bottom mitochondrion and made contact when it underwent tubulation. After the tubule retracted, the lysosome returned to its original position and fused with a larger lysosome next to it. After another 10 min of continuous imaging, we observed that a section of the mitochondrion suddenly disappeared at 16.5 min (marked by the yellow arrow). The disappearance of the mitochondrion may have been caused by membrane rupture, probably because of the influence of some external factors, such as pressure imbalance inside and outside the mitochondrion. The whole frames are shown in Supplementary Video 8. We performed 2D morphological analysis of the mitochondria and lysosomes in the two white rectangular labeled regions in Fig. S22a, in which the count, total area, mean area, mean perimeter, and mean aspect ratio of the mitochondria and lysosomes are shown (**Figure S22**, **d**). The results show that all those parameters of the mitochondrial structure are greater than those of the lysosome. More importantly, no significant changes were observed in lysosomal structure throughout the imaging process, while the opposite was true for the mitochondria, indicating that the mitochondrial structure changes dramatically in different states of cellular life activity. Figure S22e shows the curves of branches, branch junction, and branch end points per mitochondrion in full-field phasor-FSTM images over the imaging time. It can be seen the branches, junctions, and end points of the mitochondria increased significantly during the two active phases, which indicates the processes of mitochondrial fusion and/or tubulation.

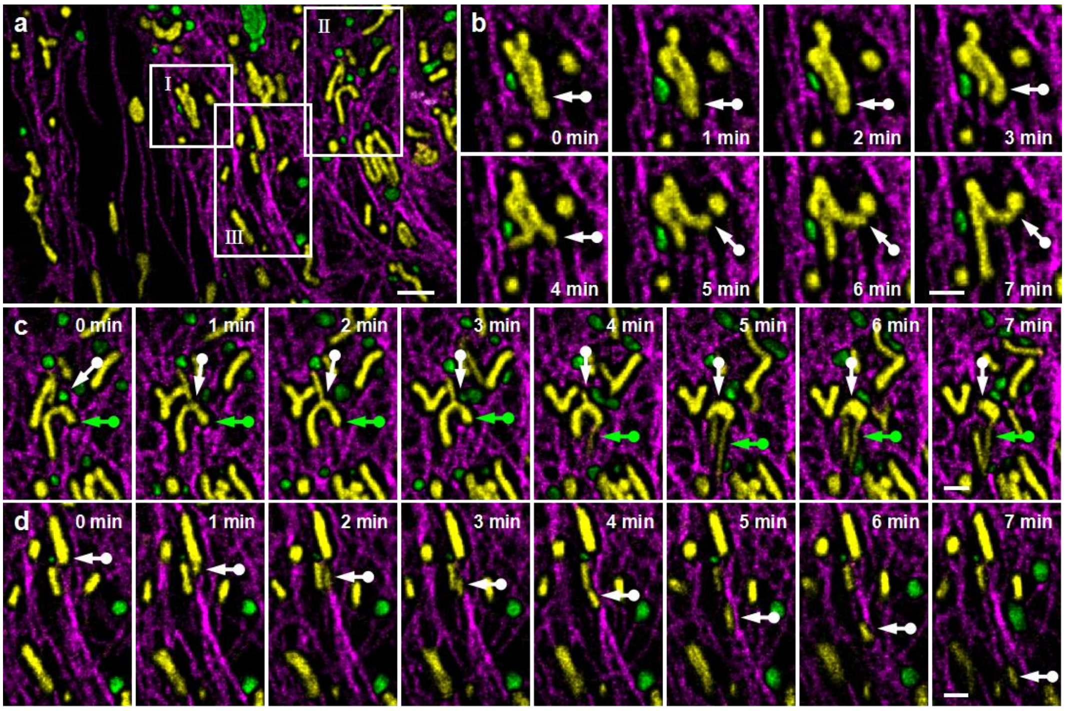

Figure S23 Long-term three-color phasor-FSTM imaging. a Three-color phasor-FSTM imaging of mitochondria and lysosomes in a large field of view . **b**-**d** The processes of mitochondrial fusion and tubulation, as well as an MDV budding off mitochondria and hitchhiking along microtubules. Scale bars, 2 μm (a, b) and 1 μm (c, d)

The function of microtubules is crucial because they are responsible for various kinds of movements in all eukaryotic cells. We made an initial study of the interaction among three organelles using long-term three-color phasor-FSTM imaging, i.e., mitochondria, lysosomes, and microtubules (**Figure S23**, **a**). The processes of mitochondrial fusion and mitochondrial tubulation were observed during the imaging course of 7 min (**Figure S23**, **b** and **c**). Interestingly, a mitochondrion has two tubules protruding from each end, and one of them is detached from the main body. In the other case, a mitochondrial-derived

vesicle (MDV) budding off a mitochondrion hitchhikes and moves about 5 μm along microtubules (**Figure S23**, **d**). More frames (8 s/frame, with 52 s dark recovery) are shown in Supplementary Video 9. All of the interactions in this paper prove once again that the living cell is a self-organizing, self-repairing, environmentally responsive machine of staggering complexity.

Comparison with other SRM techniques

In existing multicolor imaging, especially multicolor SRM imaging, spectral separation remains the most important strategy. It expands the number of discernible structures by incorporating optoelectronic components, primarily laser sources and detectors. It is well known that using multiple lasers and detectors increases the complexity of the imaging system and can have an impact on its stability and cost. We have made comparisons with conventional multicolor super-resolution fluorescence microscopy technologies (**Figure S24**). Multicolor imaging for STED requires additional excitation/depletion lasers and detectors, as studies [33–38] have shown. Multicolor STED imaging with up to five colors can be achieved by combining FLIM technology with a single depletion laser and a multi-wavelength excitation laser [39]. SIM and SMLM technologies, on the other hand, do not require a depletion laser due to their different principles; however, multicolor imaging is still dependent on the combination of distinct excitation lasers and detectors [40–46]. Our FSTM system, which uses only a single wavelength laser, a PMT detector, and a TCSPC module, when combined with our proposed phasor analysis scheme, supports up to four-color super-resolution imaging, presenting a model capable of achieving simplicity, flexibility, and affordability.

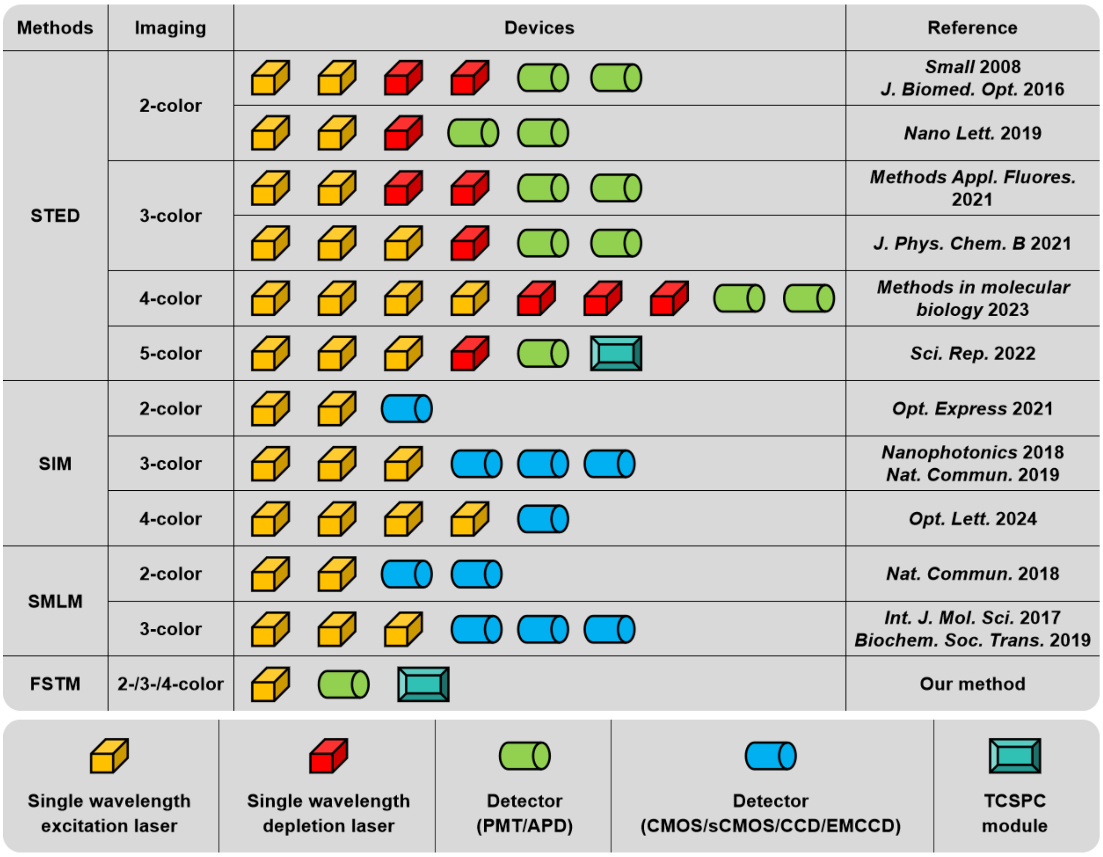

Figure S24 Comparisons with conventional multicolor SRM technologies in the number of important optoelectronic devices such as laser sources and detectors

The phasor plots at different photon number ratios

We investigated the impact of varying photon number ratios on phasor plots in two-color imaging (**Figure S25**). Before structure separation, we identified three distinct cell regions with varying proportions of microtubules and mitochondria, as shown in Figures S25a and S25b. Phasor analysis of these regions yielded distinct phasor plots (**Figure S25**, **c**), demonstrating the variation in photon distribution. Despite the inherent photon ratio differences, the phasor method allowed for excellent discrimination between structures in these regions (as shown in **Figure S25**, **d**). Furthermore, by quantifying the photon number ratio and determining the maximum photon count for each structure (**Figure S25**, **e**), we gained insight into the dynamic changes in photon ratios across cellular regions. It is important to note that the coordinates of the pixel with the highest photon number in a phasor plot are not unaffected by the number of photons; instead, the coordinates of each pixel in the phasor space are independent of the number of photons.

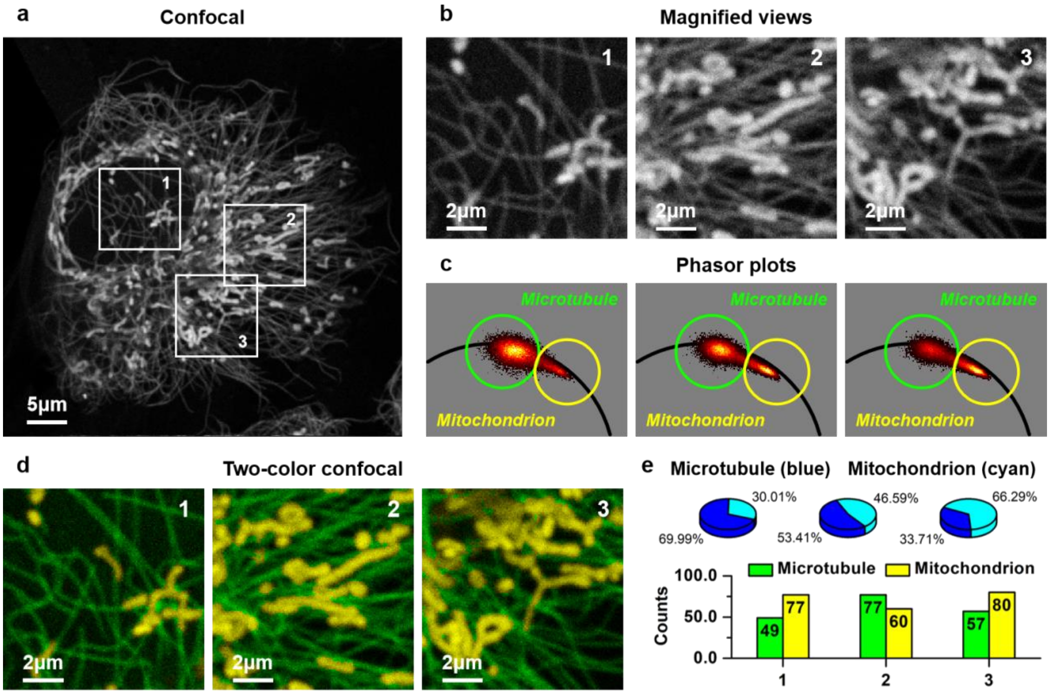

Figure S25 The phasor plots at different photon number ratios in two-color imaging. a Confocal image of a single HeLa cell consisting of mitochondrion and microtubule structures. **b** Magnified views of the three white squares in **a**. **c** Phasor plots of three images in **b**. **d** Two-color confocal images after phasor analysis (Microtubule: green; mitochondrion: yellow). **e** The ratio of the number of photons of the two structures and the maximum number of photons of each structure

Supplementary Bibliography

1. Wang, L., Li, J., Chen, Y., Guo, Y., Yang, Z., Weng, X., Yan, W. & Qu, J. Implementation of a fluorescence spatiotemporal modulation super-resolution microscope. *Optics Letters* **47**, 581–584 (2022).
2. Digman, M. A., Caiolfa, V. R., Zamai, M. & Gratton, E. The phasor approach to fluorescence lifetime imaging analysis. *Biophysical Journal* **94**, L14–L16 (2008).
3. Lou, J., Scipioni, L., Wright, B. K., Bartolec, T. K., Zhang, J., Masamsetti, V. P., Gaus, K., Gratton, E., Cesare, A. J. & Hinde, E. Phasor histone FLIM-FRET microscopy quantifies spatiotemporal rearrangement of chromatin architecture during the DNA damage response. *Proceedings of the National Academy of Sciences of the United States of America* **116**, 7323–7332 (2019).
4. Scipioni, L., Gratton, E., Diaspro, A. & Lanzanò, L. Phasor analysis of local ICS detects heterogeneity in size and number of intracellular vesicles. *Biophysical Journal* **111**, 619–629 (2016).
5. Ranjit, S., Malacrida, L., Jameson, D. M. & Gratton, E. Fit-free analysis of fluorescence lifetime imaging data using the phasor approach. *Nature Protocols* **13**, 1979–2004 (2018).
6. Scipioni, L., Rossetta, A., Tedeschi, G. & Gratton, E. Phasor S-FLIM: a new paradigm for fast and robust spectral fluorescence lifetime imaging. *Nature Methods* **18**, 542–550 (2021).
7. Fereidouni, F., Bader, A. N. & Gerritsen, H. C. Spectral phasor analysis allows rapid and reliable unmixing of fluorescence microscopy spectral images. *Optics Express* **20**, 12729–12741 (2012).
8. Cutrale, F. et al. Hyperspectral phasor analysis enables multi-plexed 5D in vivo imaging. *Nature Methods* **14**, 149–152 (2017).
9. Hedde, P. N., Cinco, R., Malacrida, L., Kamaid, A. & Gratton, E. Phasor-based hyperspectral snapshot microscopy allows fast imaging of live, three-dimensional tissues for biomedical applications. *Communications Biology* **4**, 721 (2021).
10. Stringari, C., Cinquin, A., Cinquin, O., Digman, M. A., Donovan, P. J. & Gratton, E. Phasor approach to fluorescence lifetime microscopy distinguishes different metabolic states of germ cells in a live tissue. *Proceedings of the National Academy of Sciences of the United States of America* **108**, 13582–13587 (2011).
11. Lanzanò, L., Hernάndez, I. C., Castello, M., Gratton, E., Diaspro, A. & Vicidomini, G. Encoding and decoding spatio-temporal information for super-resolution microscopy. *Nature Communications* **6**, 6701 (2015).
12. Wang, L., Chen, B., Yan, W., Yang, Z., Peng, X., Lin, D., Weng, X., Ye, T. & Qu, J. Resolution improvement in STED super resolution microscopy at low power using a phasor plot approach. *Nanoscale* **10**, 16252–16260 (2018).
13. Tortarolo, G., Sun, Y., Teng, K., Ishitsuka, Y., Lanzanό, L., Selvin, P. R., Barbieri, B., Diaspro, A. & Vicidomini, G. Photon-separation to enhance the spatial resolution of pulsed STED microscopy. *Nanoscale* **11**, 1754–1761 (2019).
14. O'Connor, D. V., Phillips, D. Time-correlated single photon counting, Academic Press, London (1984).
15. Becker, W. The bh TCSPC handbook. 10th edition, pp. 477–481 (2023).
16. Vicidomini, G., Moneron, G., Eggeling, C., Rittweger, E. & Hell, S. W. STED with wavelengths closer to the emission maximum. *Optics Express* **20**, 5225–5236 (2012).
17. Wang, L., Chen, Y., Guo, Y., Xie, W., Yang, Z., Weng, X., Yan, W. & Qu, J. Low-power STED

nanoscopy based on temporal and spatial modulation. *Nano Research* **15**, 3479–3486 (2021).

18. Descloux, A., Grußmayer, K. S. & Radenovic, A. Parameter-free image resolution estimation based on decorrelation analysis. *Nature Methods* **16**, 918–924 (2019).
19. Berman, S. B., Chen, Y., Qi, B., McCaffery, J. M., Rucker, III, E. B., Goebbels, S., Nave, K. A., Arnold, B. A., Jonas, E. A., Pineda, F. J. & Hardwick, J. M. Bcl-x_L increases mitochondrial fission, fusion, and biomass in neurons. *Journal of Cell Biology* **184**, 707–719 (2009).
20. Brookes, P. S., Yoon, Y., Robotham, J. L., Anders, M. W. & Sheu, S. S. Calcium, ATP, and ROS: a mitochondrial love-hate triangle. *American Journal of Physiology-Cell Physiology* **287**, C817–C833 (2004).
21. Chaudhry, A., Shi, R. & Luciani, D. A pipeline for multidimensional confocal analysis of mitochondrial morphology, function, and dynamics in pancreatic β-cells. *American Journal of Physiology-Endocrinology and Metabolism* **318**, E87–E101 (2020).
22. Valente, A. J., Maddalena, L. A., Robb, E. L., Moradi, F. & Stuart, J. A. A simple ImageJ macro tool for analyzing mitochondrial network morphology in mammalian cell culture. *Acta Histochemica* **119**, 315–326 (2017).
23. Nylk, J., McCluskey, K., Preciado, M. A., Mazilu, M., Yang, Z., Gunn-Moore, F. J., Aggarwal, S., Tello, J. A., Ferrier, D. E. K. & Dholakia, K. Light-sheet microscopy with attenuation-compensated propagation-invariant beams. *Science Advances* **4**, eaar4817 (2018).
24. Geijer, M. E., Moelijker, N., Zhang, G., Derr, R., Osterlund, T., Hendriks, G. & Brandsma, I. TubulinTracker, a Novel In Vitro Reporter Assay to Study Intracellular Microtubule Dynamics, Cell Cycle Progression, and Aneugenicity. *Toxicological Sciences* **186**, 288–297 (2022).
25. Choi, S. Y., Huang, P., Jenkins, G. M., Chan, D. C., Schiller, J. & Frohman, M. A. A common lipid links Mfn-mediated mitochondrial fusion and SNARE-regulated exocytosis. *Nature Cell Biology* **8**, 1255–1262 (2006).
26. Mahata, B., Mukherjee, S., Mishra, S., Bandyopadhyay, A. & Adhya, S. Functional Delivery of a Cytosolic tRNA into Mutant Mitochondria of Human Cells. *Science* **314**, 471–474 (2006).
27. Rui, Y., Wilson, D. R., Choi, J., Varanasi, M., Sanders, K., Karlsson, J., Lim, M. & Green, J. J. Carboxylated branched poly (β-amino ester) nanoparticles enable robust cytosolic protein delivery and CRISPR-Cas9 gene editing. *Science Advances* **5**, eaay3255 (2019).
28. Lin, L., Jhao, Y., Chiu, C., Sun, L., Chou, T., Shiue, C., Cheng, C. & Ma, K. Bezafibrate Exerts Neuroprotective Effects in a Rat Model of Sporadic Alzheimer's Disease. *Pharmaceuticals* **15**, 109 (2022).
29. Zhao, Y., Xie, X., Tian, L., Liu, F., Sun, Y., Lu, H., Zhao, X. & Mao, Y. MARCKSL1 interacted with F-actin to promote esophageal squamous cell carcinoma mobility by modulating the formation of invadopodia. *Cancer Medicine* **12**, 3299–3312 (2022).
30. Xydias, D., Ziakas, G., Psilodimitrakopoulos, S., Lemonis, A., Bagli, E., Fotsis, T., Gravanis, A., Tzeranis, D. S. & Stratakis, E. Three-dimensional characterization of collagen remodeling in cell-seeded collagen scaffolds via polarization second harmonic generation. *Biomedical Optics Express* **12**, 1136–1153 (2021).
31. Qiao, Q., Liu, W., Chen, J., Wu, X., Deng, F., Fang, X., Xu, N., Zhou, W., Wu, S., Yin, W., Liu, X. & Xu, Z. An Acid-regulated self-blinking fuorescent probe for resolving whole-cell lysosomes with long-term nanoscopy. *Angewandte Chemie-International Edition* **61**, e202202961 (2022).
32. Wong, Y. C., Kim, S., Peng, W. & Krainc, D. Regulation and Function of Mitochondria–Lysosome Membrane Contact Sites in Cellular Homeostasis. *Trends in Cell Biology* **29**, 500–513 (2019).

33. Meyer, L., Wildanger, D., Medda, R., Punge, A., Rizzoli, S. O., Donnert, G. & Hell, S. W. Dual-Color STED Microscopy at 30-nm Focal-Plane Resolution. *Small* **4**, 1095–1100 (2008).
34. Stephanie, S. A., Ozbay, B. N., Potcoava, M., Salcedo, E., Restrepo, D. & Gibson, E. A. Super-resolution imaging of ciliary microdomains in isolated olfactory sensory neurons using a custom two-color stimulated emission depletion microscope. *Journal of Biomedical Optics* **21**, 066017 (2016).
35. Spahn, C., Grimm, J. B., Lavis, L. D., Lampe, M. & Heilemann, M. Whole-Cell, 3D, and Multicolor STED Imaging with Exchangeable Fluorophores. *Nano Letters* **19**, 500–505 (2019).
36. Török, G., Cserép, G. B., Telek, A., Arany, D., Váradi, M., Homolya, L., Kellermayer, M., Kele, P. & Németh, K. Large Stokes-shift bioorthogonal probes for STED, 2P-STED and multi-color STED nanoscopy. *Methods and Applications in Fluorescence* **9**, 015006 (2021).
37. Pisfil, M. G., Rohilla, S., König, M., Krämer, B., Patting, M., Koberling, F. & Erdmann, R. Triple-Color STED Nanoscopy: Sampling Absorption Spectra Differences for Efficient Linear Species Unmixing. *Journal of Physical Chemistry B* **125**, 5694–5705 (2021).
38. Albus, C. A., Berlinguer-Palmini, R., Chrzanowska-Lightowlers, Z. M. & Lightowlers, R. N. Four-Color STED Super-Resolution RNA Fluorescent In Situ Hybridization and Immunocytochemistry to Visualize Mitochondrial mRNAs in Context with Mitochondrial Ribosomes. *Methods in Molecular Biology* **2661**, 303–316 (2023).
39. Pisfil, M. G., Nadelson, I., Bergner, B., Rottmeier, S., Thomae, A. W. & Dietzel, S. Stimulated emission depletion microscopy with a single depletion laser using five fluorochromes and fluorescence lifetime phasor separation. *Scientific Reports* **12**, 14027 (2022).
40. Lachetta, M., Wiebusch, G., Hübner, W., Esch, J. S. A., Huser, T. & Müller, M. Dual color DMD-SIM by temperature-controlled laser wavelength matching. *Optics Express* **29**, 39696–39708 (2021).
41. Opstad, I. S., Wolfson, D. L., Oie, C. I. & Ahluwalia, B. S. Multi-color imaging of sub-mitochondrial structures in living cells using structured illumination microscopy. *Nanophotonics* **7**, 935–947 (2018).
42. Markwirth, A., Lachetta, M., Mönkemöller, V., Heintzmann, R., Hübner, W., Huser, T. & Müller, M. Video-rate multi-color structured illumination microscopy with simultaneous real-time reconstruction. *Nature Communications* **10**, 4315 (2019).
43. Gong, D., Cai, C., Strahilevitz, E., Chen, J. & Scherer, N. F. Easily scalable multi-color DMD-based structured illumination microscopy. *Optics Letters* **49**, 77–80 (2024).
44. Lu, X., Nicovich, P. R., Zhao, M. C., Nieves, D. J., Mollazade, M., Vivekchand, S. R. C., Gaus, K. & Gooding, J. J. Monolayer surface chemistry enables 2-colour single molecule localisation microscopy of adhesive ligands and adhesion proteins. *Nature Communications* **9**, 3320 (2018).
45. Virant, D., Turkowyd, B., Balinovic, A. & Endesfelder, U. Combining Primed Photoconversion and UV-Photoactivation for Aberration-Free, Live-Cell Compliant Multi-Color Single-Molecule Localization Microscopy Imaging. *Internation Journal of Molecular Sciences* **18**, 1524 (2017).
46. Vojnovic, I., Winkelmeier, J. & Endesfelder, U. Visualizing the inner life of microbes: practices of multi-color single-molecule localization microscopy in microbiology. *Biochemical Society Transactions* **47**, 1041–1065 (2019).
